# Prolonged exposure to high fluoride levels during adolescence to adulthood elicits molecular, morphological, and functional impairments in the hippocampus

**DOI:** 10.1038/s41598-023-38096-8

**Published:** 2023-07-08

**Authors:** Leonardo Oliveira Bittencourt, Aline Dionizio, Maria Karolina Martins Ferreira, Walessa Alana Bragança Aragão, Sabrina de Carvalho Cartágenes, Bruna Puty, Cristiane do Socorro Ferraz Maia, Fatemeh Vida Zohoori, Marília Afonso Rabelo Buzalaf, Rafael Rodrigues Lima

**Affiliations:** 1 Laboratory of Functional and Structural Biology, Institute of Biological Sciences, Federal University of Pará, Augusto Corrêa street n. 01, Guamá, Belém, Pará 66075-110 Brazil; 2 Department of Biological Sciences, Bauru Dental School, University of São Paulo, Bauru, São Paulo Brazil; 3 Laboratory of Inflammation and Behavior Pharmacology, Faculty of Pharmacy, Institute of Health Sciences, Federal University of Pará, Belém, Pará Brazil; 4 School of Health and Life Sciences, Teesside University, Middlesbrough, United Kingdom

**Keywords:** Biochemistry, Cell biology, Environmental sciences

## Abstract

Fluoride is added to water due to its anticariogenic activity. However, due to its natural presence in soils and reservoirs at high levels, it could be a potential environmental toxicant. This study investigated whether prolonged exposure to fluoride from adolescence to adulthood—at concentrations commonly found in artificially fluoridated water and in fluorosis endemic areas—is associated with memory and learning impairments in mice, and assessed the molecular and morphological aspects involved. For this endeavor, 21-days-old mice received 10 or 50 mg/L of fluoride in drinking water for 60 days and the results indicated that the increased plasma fluoride bioavailability was associated with the triggering of short- and long-term memory impairments after high F concentration levels. These changes were associated with modulation of the hippocampal proteomic profile, especially of proteins related to synaptic communication, and a neurodegenerative pattern in the CA3 and DG. From a translational perspective, our data provide evidence of potential molecular targets of fluoride neurotoxicity in the hippocampus at levels much higher than that in artificially fluoridated water and reinforce the safety of exposure to low concentrations of fluoride. In conclusion, prolonged exposure to the optimum fluoride level of artificially fluoridated water was not associated with cognitive impairments, while a higher concentration associated with fluorosis triggered memory and learning deficits, associated with a neuronal density reduction in the hippocampus.

## Introduction

Fluoride is a chemical element naturally found in the environment that has high electronegativity and a great capacity to form compounds by binding to other chemical elements, forming organic and inorganic compounds such as hydrofluoric acid (HF) and sodium fluoride (NaF). In addition, fluoride is used in the pharmaceutical industry and aluminum manufacturing processes and is also present in waste product of ceramic production and coal burning. Due to its anticariogenic activity, it is also added to the public domestic water supply^1–3^.

Since 1945, cities around the world have fluoridated their public water supply^4^. The World Health Organization (WHO) has determined that the optimum safe and effective fluoride level against dental caries is 0.5–1.0 mg/L^5^, ranging from 0.7 to 1.2 mg/L depending on geographical conditions. Although the WHO has recommended the optimum fluoride level, it has stated that each area must consider other sources that would influence total daily fluoride intake^6^. However, several questions have been raised about safety and ethical issues involving artificially fluoridated water, which is often erroneously associated with high levels found in water for human consumption due to environmental pollution caused by industrial activity or even due to high natural fluoride levels, which may cause fluorosis and other possible neurological disorders^7,8^.

Several observational studies and systematic reviews have reported an association between exposure to fluoride and its impacts on cognitive functions in developing organisms^9,10^. However, a recently published systematic review by our group^11^ pointed out that the level considered optimum by the WHO is not associated with neurological disorders, whereas higher levels may be associated with lowering the intelligence quotient. To address this issue, we used an experimental design to investigate the association of a low fluoride dose, considered representative of artificial fluoridation of the domestic water supply, and a high fluoride dose, representative of fluoride endemic regions where fluorosis is common, based on molecular, morphological and functional aspects in mice exposed from adolescence to adulthood^12–16^. This window of exposure makes this investigation even more relevant due to the widespread knowledge that at this stage, the central nervous system (CNS) is still developing, presenting similar neurodevelopment events that occur in human children, adolescents and young adults. Thus, we aimed to answer two main questions: 1) does prolonged exposure to low and/or high fluoride concentrations impair memory and learning in mice? 2) what are the proteins involved in fluoride-induced hippocampal neurotoxicity? To answer these questions, we examined the impacts of fluoride on cognition in mice through adolescence and adulthood. We also evaluated changes in the hippocampal proteomic profile and morphology that could be involved in the outcomes found, by comparing the groups in a dose–response model perspective.

## Materials and methods

### Experimental groups and exposure protocol

All the procedures were performed after ethics committee on the use of experimental animals’ approval, under protocol no 2422071217 (Comitê de Ética no Uso de Animais da Universidade Federal do Pará CEUA-UFPA). All the procedures followed the ARRIVE guidelines. A total of 42 male mice (*Mus musculus*) at 21 days of age and a body mass of approximately 10 g were randomly divided into three groups of 14 animals each. The mice were housed in collective cages and fed with food and water ad libitum in a climate-controlled room, with a 12 h light/dark cycle. The experimental protocol was performed according previous publication^15,17,18^, which consists of dividing the animals into three groups: Control, 0 mg/L of fluoride; 10 mg/L, exposed through drinking-water to a solution containing 10 mg/L of fluoride; 50 mg/L, exposed through drinking-water to a solution containing 50 mg/L of fluoride. All groups were exposed for 60 days and the NaF salt was solubilized in ultrapure water, the same used for control group. These concentrations are justified by the need for fluoride administration to be 4–5 times higher in rodents in order to reach plasma concentrations and clinical manifestations similar to those found in humans, and represent artificially fluoridated drinking water and water from fluoride endemic areas^18,19^. No inclusion and exclusion criteria were considered before and after animal experimentation, besides the animal characteristics mentioned above.

### Behavioural assessment

After exposure to fluoride, the animals were selected randomly and taken to the behavioural testing room with attenuated sound and controlled lighting and temperature. The animals were habituated to the environment for a minimum of 1 h prior the beginning of the behavioural tests described below.

The inhibitory avoidance test uses an aversive stimulus as a factor to examine changes in short and long-term memory^20^. This test is completely described elsewhere^21^. Briefly, it is performed in two consecutive days: In the first day the animals are placed in a secure platform in the apparatus and when they step-down towards a metal grid, an aversive stimulus (0.4 mA electric shock) was applied for one second. Then, to evaluate the short-term memory, 90 min after the shock the animals were re-exposed to the apparatus, but no stimulus was applied. In this second day, the same procedure was performed to evaluate long-term memory. The ability of learning and remembering that the metal grid of the apparatus is capable of triggering an aversive stimulus was observed by the latency time to step-down from a secure platform to the grid i.e., as higher the latency time, more preserved are the cognitive functions.

The Morris Water Maze was used to assess learning and spatial memory and followed a previously published protocol^22,23^. The task comprises a circular water tank filled with water (25 °C) up to 30 cm, divided into four equal quadrants (Q1–Q4). A 29 cm high platform (10 cm^2^) made of blue acrylic was placed in the centre of the target quadrant (Q4) for the training session and removed from the pool 24 h after training. Each animal was subjected to four consecutive training sessions with 5 min between each session; after each session, the animal remained on the platform for 20 s. The time spent in each quadrant and the time needed to find the platform were recorded. On the test day (day 2), the animals were placed in the pool, without the platform, and allowed to explore the area for 60 s. The time needed to reach Q4, the quadrant in which the platform was on the training day, and the time spent in Q4 were recorded and analysed.

### Sample collection

A total of 18 animals (6 per group) were used for plasma fluoride level determination and proteomic analyses. Samples were collected after anaesthetic induction with a solution of ketamine hydrochloride (90 mg/kg) and xylazine hydrochloride (10 mg/kg). Then, the blood was collected through cardiac puncture with and centrifuged at 3000 rpm for 10 min to collect the plasma. Subsequently, the brain was removed and the hippocampal formation was dissected in ice-cold phosphate buffered saline (PBS) and immediately frozen in liquid nitrogen and kept at − 80 °C.

### Fluoride level assessment

In order to validate the exposure protocol, i.e., to confirm that different fluoride levels in drinking generated different ion bioavailabilities in plasma, the fluoride concentrations in the plasma were determined in duplicate after HMDS-facilitated diffusion for 12 h using a fluoride-specific electrode (Orion Research, Model 9409) and a miniature calomel electrode (Accumet, 13-620-79), coupled to a potentiometer (Orion Research, Model EA 940), as previously described^24,25^. Millivoltage readings were converted to μg of fluoride based on a standard curve.

### Proteomic and bioinformatic analyses

A total of 6 animal per group was used in these analyses. The methodology was carried out in biological triplicate, after pooling two animals from the same group into one single sample. After, the samples were submitted to a cryogenic mill, followed by protein extraction by buffer lysis under constant stirring and 4 °C. Then, a standardized protein concentration was determined by Bradford’s method (1 μg/μL) and a fixed protein amount (50 μg) was used for the following steps: alkylation, digestion, desalting and elution. The reading and identification of the peptides was performed by using the nanoAcquity UPLC-Xevo QTof MS system (Waters Co., UK). Detailed methodology is available elsewhere^23,26^.

### Morphological analyses

After the behavioural tests, eight animals per group were assigned randomly to immunohistochemical evaluations. After anaesthesia they were perfused with heparinised saline solution (0.9%) followed by 4% paraformaldehyde in 0.2 M PBS. The brain samples were embedded in Paraplast^®^ (Sigma-Aldrich, USA) and 5 µm thickness sections from dorsal hippocampus were obtained by a microtome. For the indirect immunohistochemistry, we used the anti-NeuN antibody (1:100, Millipore). After revelation with diaminobenzidine, the positive cells were counted in the hippocampal CA1, CA3, dentate gyrus (DG) and hilus regions as previously described^23^. Photomicrographs were obtained by DS-Fi3 microscope camera attached to the Nikon Eclipse Ci H550s bright field microscope.

### Statistical analyses

For data analyses, one-way or two-way analysis of variance (ANOVA) was performed depending on the variable, followed by Tukey’s post hoc test with *p* < 0.05 as significant difference. The results are expressed as mean ± standard error of mean (SEM). In the proteomic analysis, the difference in expression between groups was obtained by using the ProteinLynx GlobarSERVER software, considering *p* < 0.05 for downregulated proteins and 1—*p* > 0.95 for upregulated proteins. The results are expressed as mean ± standard error of mean (SEM).

**Figure 1:**
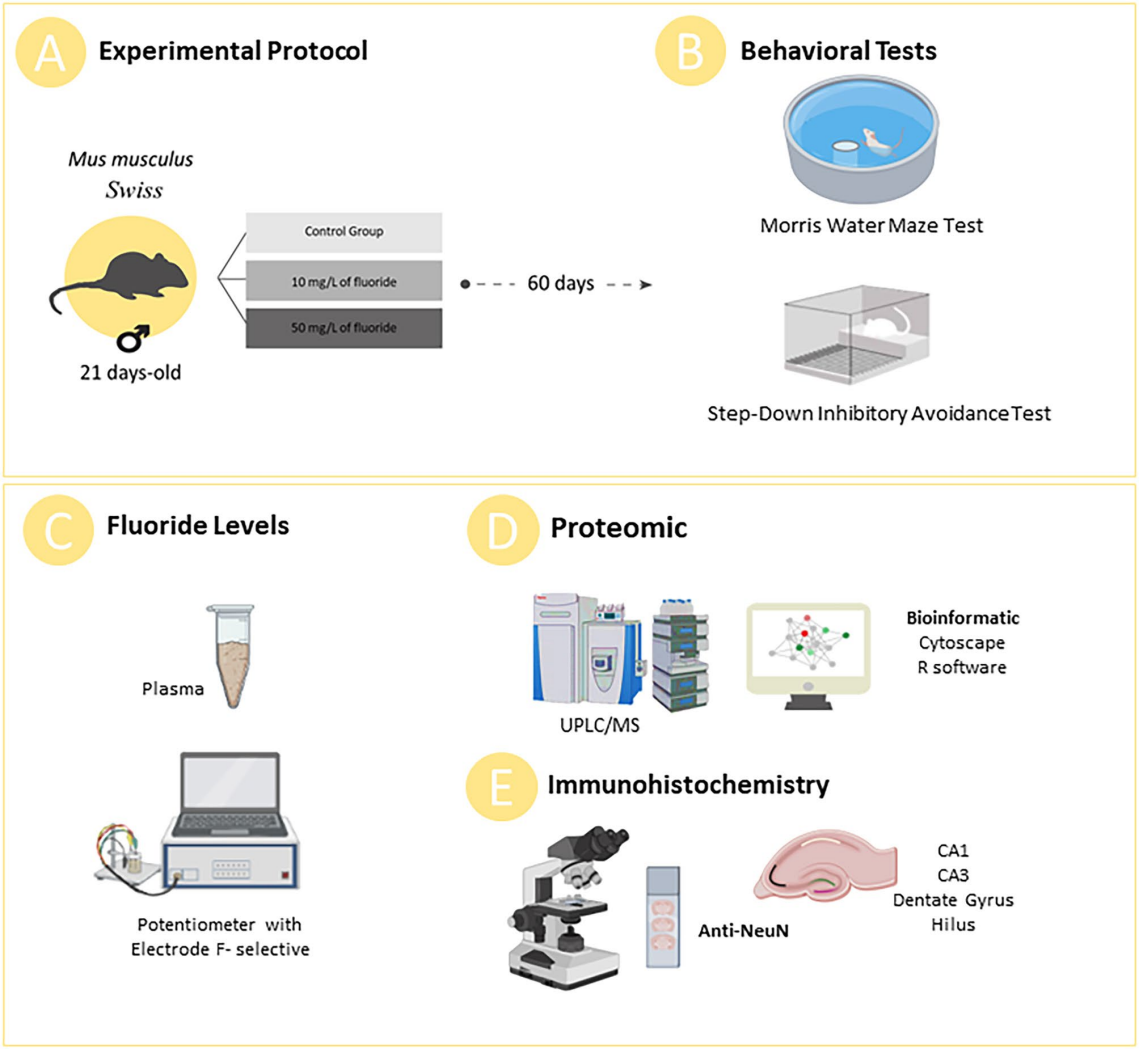
Methodological scheme summarizing the experimental and analytical steps of the study. In (**A**) the protocol of fluoride exposure during 60 days in male mice and groups division. In (**B**) behavioral assessments performed after exposure period to analyze cognitive functions. After euthanasia, blood and hippocampus were collect to assess the plasma fluoride levels (**C**) and global proteomic profile of the hippocampus (**D**). A set of animals were perfused for immunohistochemical analyses by investigating anti-NeuN immunostaining in the hippocampal regions for cell density determination.

Methodological steps are summarized in Fig. 1.

### Ethical approval

All the procedures were performed after ethics committee on the use of experimental animals’ approval, under protocol nº 2,422,071,217 (Comitê de Ética no Uso de Animais da Universidade Federal do Pará CEUA-UFPA). All the procedures followed the ARRIVE guidelines.

## Results

### Increased fluoride bioavailability did not impact body weight gain

**Figure 2:**
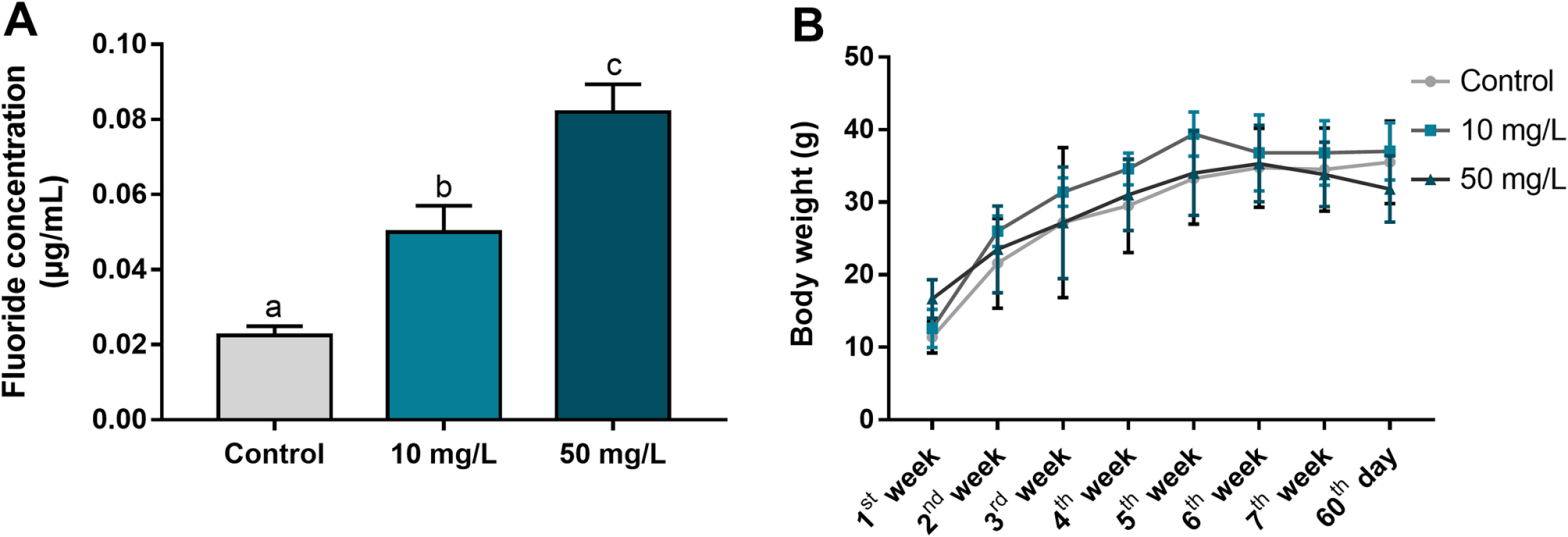
The effects of 60 days of fluoride exposure (10 or 50 mg/L) from adolescence to adulthood on the plasma fluoride level of mice (**A**) and body weight (g) during the experimental period (**B**). The data are presented as mean ± standard error of the mean (SEM). Statistical analysis: in A, one-way analysis of variance (ANOVA) with Tukey’s post hoc test; in (**B**), two-way ANOVA. Different letters indicate a significant difference (*p* < 0.05).

The systemic fluoride administration was validated by measuring the plasma fluoride levels, which confirmed the exposure by the increasing ion bioavailability in a dose-dependent manner (*p* = 0.0002). The mean ± SEM fluoride concentration was 0.02 ± 0.002 μg/mL for the control group, 0.05 ± 0.007 μg/mL for the 10 mg/L group and 0.08 ± 0.007 μg/mL for the 50 mg/L group (Fig. 2A). Body weight gain during the experimental period was not affected by fluoride exposure (*p* > 0.05, Fig. 2B).

### Prolonged exposure to fluoride modulated the hippocampal proteomic profile

The hippocampal proteomic profile of mice exposed to both fluoride concentrations were changed significantly (Table 1).

**Table 1:** Quantitative distribution of proteins with different status of regulation among the group comparison.

Comparison	Up-regulated	Down-regulated	Exclusive in first group	Exclusive in second group
10 mg/L versus Control	126	65	136	117
50 mg/L versus Control	121	23	140	104
50 mg/L versus 10 mg/L	86	138	109	87

Bioinformatic analysis of biological processes in which the proteins are involved showed a similar profile considering the group comparisons as summarised in Table 2. The most impacted processes are related to morphological and energy metabolism aspects. The complete list of biological processes is presented in Supplementary Table 1 and the complete lists of proteins of each comparison are presented in Supplementary Tables 2–4. Some relevant proteins were highlighted in Table 3 for further discussion.

**Table 2:** List of biological processes based on Gene Ontology according to the hippocampal proteome in each group comparison.

Group comparison	Biological process	Number of genes (%)
10 mg/L × Control	Axon guidance	13.9
	Regulation of axonogenesis	12.6
	Dendritic spine morphogenesis	8.8
	Mitochondrial ATP synthesis coupled proton transport	7.5
	Glycolytic process through fructose-6-phosphate	6.3
+ 16 biological processes		
50 mg/L × Control	Axon guidance	13.5
	Regulation of axonogenesis	10.6
	Mitochondrial ATP synthesis coupled proton transport	6.7
	Regulation of dendritic spine development	6.7
	Transcription corepressor activity	5.8
+ 22 biological processes		
50 mg/L × 10 mg/L	Regulation of axonogenesis	12.6
	Axon guidance	9.5
	Positive regulation of dendritic spine development	9.5
	Arp2/3 complex-mediated actin nucleation	7.4
	Mitochondrial ATP synthesis coupled proton transport	6.3
+ 20 biological processes

**Table 3:** List of proteins highlighted and their respective fold changes in the comparison among groups.

Accession ID^a^	Protein description	Fold change		
		10 mg/L versus control	50 mg/L versus control	50 mg/L versus 10 mg/L
P68033	Actin, alpha cardiac muscle 1	− 0.878	1.32	2.25
P68134	Actin, alpha skeletal muscle	− 0.403	1.34	2.27
P62737	Actin, aortic smooth muscle	− 0.415	1.34	2.27
P63268	Actin, gamma-enteric smooth muscle	− 0.932	1.45	2.23
P11798	Calcium/calmodulin-dependent protein kinase type II subunit alpha	− 0.487	1.46	1.82
P28652	Calcium/calmodulin-dependent protein kinase type II subunit beta	− 0.492	1.07	− 0.87
P0DP26	Calmodulin-1	− 0.741	1.77	1.57
P0DP27	Calmodulin-2	− 0.733	1.20	1.58
P0DP28	Calmodulin-3	− 0.741	1.20	1.6
Q91XM9	Disks large homolog 2	10 mg/L	–	10 mg/L
Q62108	Disks large homolog 4	10 mg/L	–	10 mg/L
P10649	Glutathione S-transferase Mu 1	1.391	−	− 0.81
P15626	Glutathione S-transferase Mu 2	2.718	2.16	–
P48774	Glutathione S-transferase Mu 5	–	1.79	–
Q80W21	Glutathione S-transferase Mu 7	2.718	2.03	–
P19157	Glutathione S-transferase P 1	10 mg/L	50 mg/L	–
P46425	Glutathione S-transferase P 2	10 mg/L	–	10 mg/L
P17879	Heat shock 70 kDa protein 1B	− 0.487	–	1.67
P16627	Heat shock 70 kDa protein 1-like	− 0.733	− 0.88	1.54
Q61316	Heat shock 70 kDa protein 4	10 mg/L	50 mg/L	–
P48722	Heat shock 70 kDa protein 4L	10 mg/L	–	–
P63017	Heat shock cognate 71 kDa protein	–	− 0.90	1.48
Q61699	Heat shock protein 105 kDa	10 mg/L	50 mg/L	–
Q9CQN1	Heat shock protein 75 kDa, mitochondrial	3.353	1.68	− 0.5
P07901	Heat shock protein HSP 90-alpha	−	1.38	− 0.68
P17156	Heat shock-related 70 kDa protein 2	− 0.463	− 0.66	1.48
Q9QYR6	Microtubule-associated protein 1A	10 mg/L	50 mg/L	− 0.66
P20357	Microtubule-associated protein 2	1.350	–	− 0.76
Q7TSJ2	Microtubule-associated protein 6	10 mg/L	–	–
P35700	Peroxiredoxin-1	10 mg/L	50 mg/L	–
Q61171	Peroxiredoxin-2	1.209	–	− 0.84
O08807	Peroxiredoxin-4	10 mg/L	50 mg/L	–
P99029	Peroxiredoxin-5, mitochondrial	–	1.09	–
P08228	Superoxide dismutase [Cu–Zn]	1.350	–	− 0.65
Q62277	Synaptophysin	− 0.543	− 0.88	1.58

^a^Accession ID based on uniprot.org database. Each subcolumn from the fold change column represents one comparison between experimental groups, where control, 10 mg/L, and 50 mg/L refer to each one of the experimental groups. The negative values mean down-regulated proteins, and the positive values, up-regulated proteins. The fold changes filled with the group’s identification mean exclusive regulation in that specific group in the correspondent comparison. Those fold changes filled with a minus sign (–) mean no significative modulation in the correspondent comparison.

**Figure 3:**
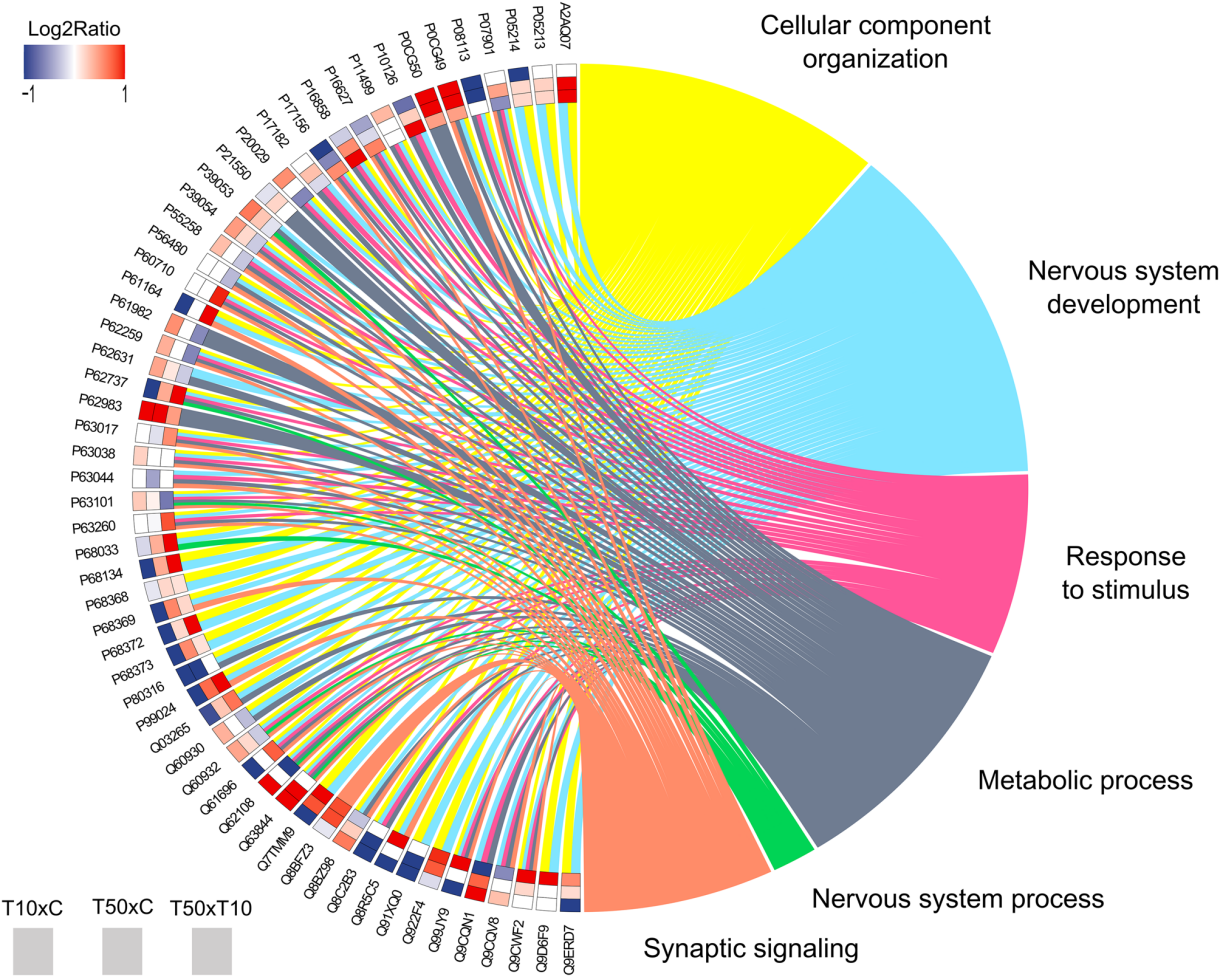
Circos plot of the protein–protein networks in the hippocampus of mice exposed to 10 or 50 mg/L of fluoride. The networks are associated with biological processes including cellular component organization (yellow), nervous system development (light blue), response to stimulus (pink), metabolic process (greyish blue), nervous system process (green) and synaptic signalling (beige), based on Gene Ontology annotations. Each protein is described with its respective UniProt accession ID, and each coloured rectangle indicates a different comparison, with a log2ratio ranging from − 1 to 1.

ORA (Fig. 3) showed the interaction of 59 proteins categorised in six main biological processes according to GO, namely cellular component organisation, nervous system development, response to stimulus, metabolic process, nervous system process and synaptic signalling.

### Prolonged exposure to a high fluoride concentration caused a reduction on neuron density in the hippocampus

**Figure 4:**
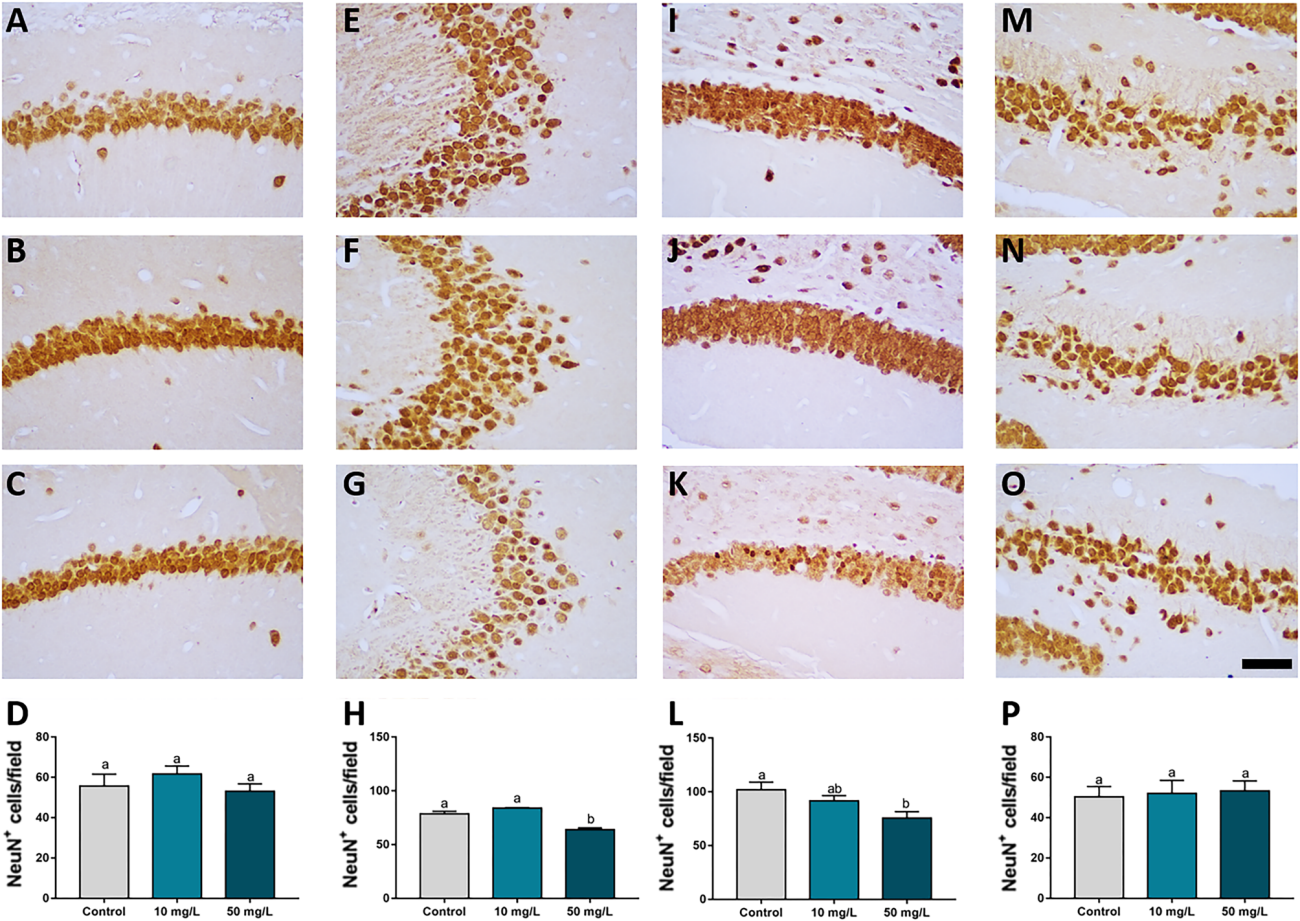
The effects of fluoride exposure from adolescence to adulthood on the mature neuronal density in mouse hippocampus. NeuN immunohistochemistry in the CA1 (**A**–**D**), CA3 (**E**–**H**), dentate gyrus (**I**–**L**) and hilus (**M**–**P**) of mouse hippocampus. The data are presented as the mean ± standard error of the mean (SEM) of the neuronal density. Different letters indicate a significant difference (*p* < 0.05, one-way analysis of variance with Tukey’s post hoc test). The scale bar is 50 µm.

Exposure to 50 mg/L of fluoride for 60 days reduced the mature neuronal density in the CA3 when compared with the control and 10 mg/L groups (adj. *p* value < 0.0001), and in DG when compared with the control group (adj. *p* value = 0.02). No significant changes were observed in the CA1 and hilus (*p* > 0.05; Fig. 4) in the 50 mg/L group, and no difference was found in any hippocampal area of animals from the 10 mg/L group.

### Prolonged exposure to a high fluoride concentration impaired cognition

**Figure 5:**
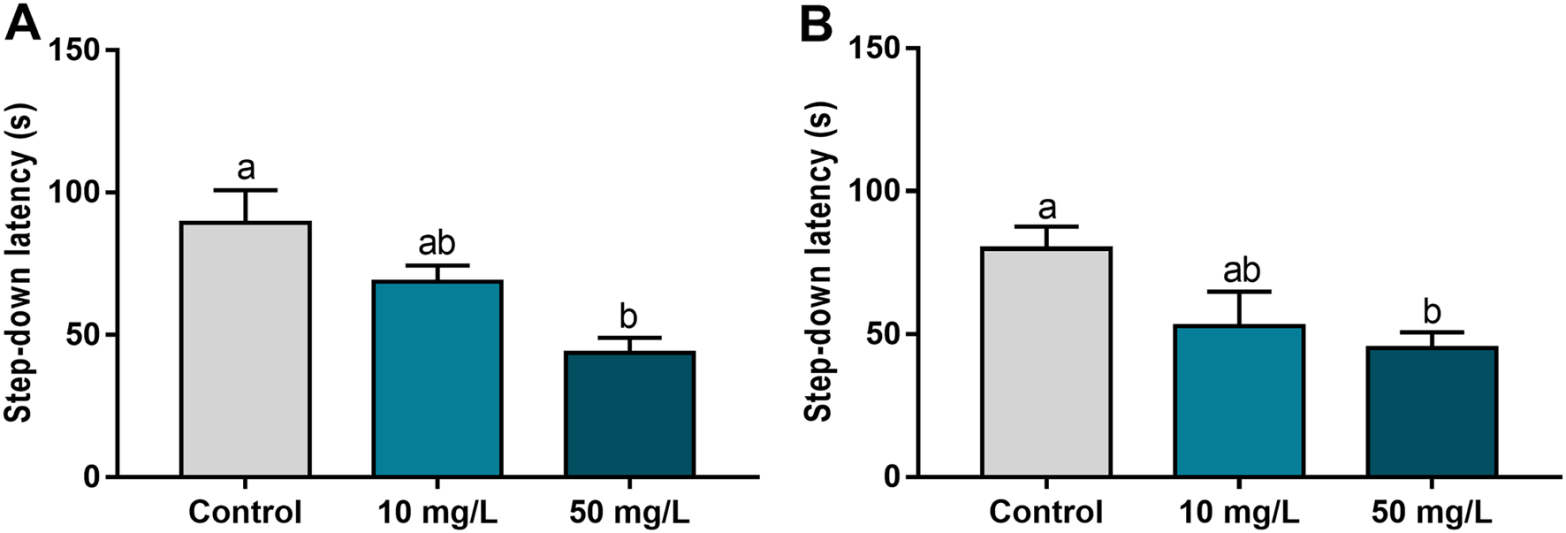
The effects of fluoride exposure from adolescence to adulthood on step-down inhibitory avoidance test performance of mice. (**A**) and (**B**) show the step-down latency (seconds) in the short-term (1.5 h) and long-term (24 h) memory assessments, respectively. Different letters indicate a significant difference (*p* < 0.05, one-way analysis of variance with Tukey’s post hoc test).

The step-down inhibitory avoidance test showed that both short-term (adj. p value = 0.0005) and long-term (adj. *p* value = 0.03) memory were impaired after exposure to 50 mg/L of fluoride (Fig. 5). There was no impairment observed in the 10 mg/L group (*p* > 0.05).

**Figure 6:**
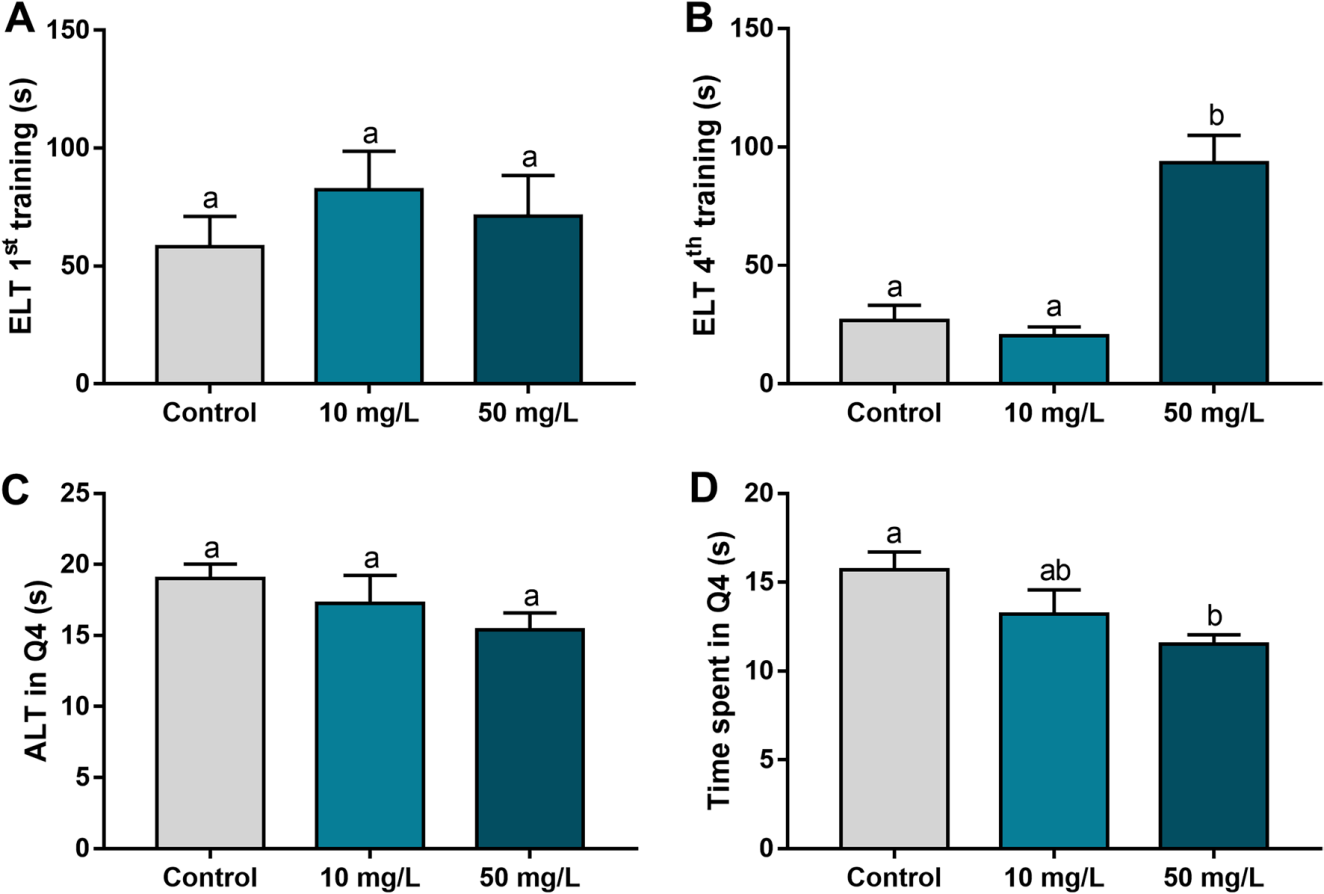
The effects of fluoride exposure from adolescence to adulthood on Morris’s water maze performance of mice. (**A**) and (**B**) show the escape latency time (ELT, seconds) in the first and fourth training sessions, respectively. (**C**) shows the arrival latency time (ALT, seconds) in the target quadrant (Q4). (**D**) shows the time spent (seconds) in the target quadrant (Q4). Different letters indicate a significant difference (*p* < 0.05, one-way analysis of variance with Tukey’s post hoc test).

Regarding spatial memory, during the training phase, the animals in the 50 mg/L group showed poorer performance compared with the control and 10 mg/L groups (adj. *p* value < 0.0001), requiring more time to reach the target quadrant after three training sessions (Fig. 6B). When assessing a learning parameter (Fig. 6D), prolonged exposure to 50 mg/L fluoride reduced the time spent in Q4 compared with the control group (adj. *p* value = 0.01).

## Discussion

This study evidenced that prolonged exposure of mice from adolescence to adulthood to a high fluoride concentration (50 mg/L) could impair cognitive functions associated with the hippocampus. The results indicate that the short- and long-term memory impairment is the result of a reduction on neuron cells density pattern in the CA3 and DG of the hippocampus, besides an intense modulation of the hippocampal proteomic profile of proteins related to synaptic function, neuroplasticity and energy metabolism. No functional impairments were observed in mice exposed to the low fluoride concentration, even with increased fluoride bioavailability and proteome modulation.

We designed our experimental model based on three main points: the age of the mice, with fluoride exposure beginning at the age of 21 days; using fluoride concentrations with translational relevance, because the lower one (10 mg/L) represents the levels found in artificially fluoridated water supplies, and the higher (50 mg/L) represents fluoride levels in endemic regions of fluorosis^18^; and the prolonged exposure model, adapted to the rodent’s metabolism^19^, that lasts from adolescence to the adult stage. Thus, to validate our exposure model, we assessed the plasma fluoride levels: the plasma fluoride bioavailability increased in the exposed animals. Our findings are consistent with previous publications that also evidenced damage to the hippocampus of rats exposed during the intrauterine and lactational periods^14^, and in the cerebellum^13^ and salivary glands^17,27^ of adult mice.

It is well known that the mammalian brain continues to develop even after birth; hence, the CNS is susceptible to xenobiotic damage during this period^28^. From 21 to 60 days of life, the mouse brain undergoes several modifications and reaches total maturation. As reviewed by Semple et al.^29^, this period corresponds to the age of 2–20 years in humans, due the similarities of the developmental events that occur in both species, such as peak synaptic density during adolescence, the myelination rate, refinement of cognitive-dependent circuitry and the adult synaptic density, among others^29^). Several observational studies have pointed to the neurodevelopmental toxic effects of fluoride exposure (for a review, see Choi et al.^9^ and Grandjean^30^). Thus, these points reinforce the representativeness of our study, especially considering the dichotomy between fluoridation of the water supply and the environmental toxicological concern, and contributes reliable evidence to the literature regarding pre-clinical outcomes associated with prolonged fluoride exposure.

We evaluated different biological organisational levels, as molecular, morphological and functional aspects from the hippocampus, a pivotal structure involved in memory and learning. Cognitive functions are defined as a set of abilities related to emotions, communication, hearing processing, decision-making, learning and memories^31^. These two last components involve complex molecular processes and anatomical structures, which allow the subdivision according to the duration (short and long-term) and content (declarative or procedural)^32^. In the step-down inhibitory avoidance task, the amygdala is an important anatomical region because of the emotional aspect of this test, but the hippocampus is pivotal for memory formation^20^. On the other hand, in the Morris' water maze, the spatial memory formation and learning processes are mediated mainly by the hippocampus^33^. Thus, we examined the hippocampus as the region of interest due to its importance in both ethological approaches. We found that exposure to a high fluoride concentration impaired short- and long-term memory based on the step-down inhibitory avoidance trial and impaired long-term memory and learning abilities based on the Morris water maze.

In order to investigate the background of such behavioral impairments, we analyzed the neuronal density of four dorsal hippocampal regions: the CA1, CA3, hilus and DG. No morphological changes were observed in the CA1 and hilus, but there was significant damage in the CA3 and DG that seems to be associated with the cognitive impairments triggered by exposure to 50 mg/L of fluoride. The DG acts as an input link between the entorhinal cortex and the hippocampus and is a site of projections from the hippocampus to other brain areas^34^. The CA3 receives inputs from the entorhinal cortex via the perforant path and from the DG by the mossy fibres^35^, which comprise the anatomical pathways involved in hippocampus-related memories.

The neuron density decrease is an important feature to be highlighted and further investigated regarding the time-window and fluoride levels exposure mainly because it is suggestive of a neurodegenerative pattern, featured in some neurological diseases such as Alzheimer’s diseases^36^. However, the cause of the density decrease must be first elucidated, whether is caused by a direct cell death already shown in previous studies^37–39^, whether is due to an impairment on the remarkable neurogenic role of hippocampus, especially the latter, considering the period of fluoride exposure ended with the CNS fully developed^29^, with 81-days-old, which corroborates with the dual suggestive pathways of our findings regarding the developmental fluoride neurotoxicity^30,40^.

Researchers have investigated the possible damage triggered by fluoride, however, they have mostly used different ages and higher fluoride concentrations. Two studies in particular assessed the global proteomic profile of rats exposed to 100 mg/L of fluoride^41^ and to 20 mg/kg/day of NaF (administered intraperitonially) for 30 days^42^. While these studies may be interesting to elucidate the mechanisms of fluoride neurotoxicity, they have important limitations regarding the translational point of view based on the dose and temporal exposure window.

Our proteomic approach showed that both fluoride concentrations modulated the hippocampal proteome. Interestingly, several proteins were common in both comparisons, but they were differentially regulated. ORA allowed us to investigate the common biological processes among the three proteomic comparisons performed. It revealed that the common proteins are mainly related to cellular component organisation, nervous system development, response to stimulus, metabolic process, nervous system process and synaptic signalling. Hence, we suggest that the different response pattern after the exposure to low and high fluoride levels may involve those biological processes.

The first set of proteins that must be discussed are involved in the response to stimulus; they play an important role in the different response patterns of the exposed groups. The heat shock proteins (HSP) are molecular chaperones involved in the processes of oxidative stress signalling, transcription processes, protein maturation and re-folding and degradation^43^. These proteins have been suggested^44^ as possible new therapeutic tools. Indeed, they are associated with neurodegenerative diseases^45^ and are part of neurodevelopment by mediating cell growth and migration, axon guidance and angiogenesis^46^. Exposure to 10 mg/L of fluoride caused downregulation of HSP 70 kDa protein 1B (P17879) and 70 kDa protein 1-like (P16627); upregulation of 75 kDa, mitochondrial (Q9CQN1) and 90 kDa-beta (P11499); and exclusive expression of 70 kDa protein 4 (Q61316), 70 kDa protein 4L (P48722) and 105 kDa (Q61699) in the exposed group. On the other hand, the 50 mg/L caused downregulation of P16627, HSP 71 kDa protein (P63017) and 70 kDa protein 2 (P17156); and upregulation of Q9CQN1 and HSP 90-alpha (P07901). This proteomic modulation may be associated with a response to the damage triggered by fluoride exposure and even compromised hippocampal development.

As mentioned before, in addition to serving as a marker of proteome protection or damage, HSP are important markers of oxidative stress^43^. From this perspective, the proteomic approach also revealed the significant modulation of antioxidant enzymes, such as upregulation of glutathione S-transferase mu 1, 2 and 7 (P10649, P15626 and Q80W21, respectively); peroxiredoxin-2 (Q61171); and superoxide dismutase [Cu–Zn] (P08228); and the exclusive expression of glutathione S-transferase P 1 and 2 (P19157 and P46425, respectively) in the 10 mg/L group. In addition, there was exclusive regulation of peroxiredoxin-1 (P35700) and peroxiredoxin-4 (O08807) in the 10 mg/L group. In the 50 mg/L, the proteins P15626 and Q80W21 were also upregulated compared with the control group, in addition to glutathione S-transferase mu 5 (P48774) and peroxiredoxin-5, mitochondrial (P99029). The proteins P35700 and O08807 were found exclusively expressed in the 50 mg/L group. These findings suggest a positive response of the enzymatic antioxidant system against reactive oxygen species in the hippocampus of mice exposed to 10 or 50 mg/L of fluoride. The changes in these proteins, and other components of the antioxidant system, are associated with fluoride-induced oxidative stress, one of the mechanisms by which fluoride exerts damage^47^).

The execution of neural functions is dependent on several structural components, molecular pathways and neurochemical communication. From this perspective, based on ORA, we highlight three biological processes: cellular component organisation, nervous system process and synaptic signalling, which presented cytoskeletal proteins, synaptosomal components and proteins related to dendritic organisation.

Cytoskeletal rearrangement plays a key role in transporting synaptic vesicles^48^), besides the maintenance of cell morphology. Structural components of both actin filaments and microtubules were altered significantly by fluoride exposure. Several tubulin alpha and beta chains (see Supplementary Tables 2, 3) were upregulated in the 10 mg/L group but downregulated in the 50 mg/L group. This may indicate an important impairment in microtubule function because this cytoskeletal component is important to maintain the cell shape, dendritic morphogenesis and the intracellular tracking of vesicles^49^). Actin filaments are well known as participants of many biological processes, such as cell migration and division, but are also determinants in the formation of dendritic spikes and consequent long-term memory consolidation. Actin filaments also participate actively in neuronal exocytosis and endocytosis^48,50,51^. Interestingly, this cytoskeletal component showed an opposite profile compared with microtubule constituents. Several actin isoforms were down-regulated in the 10 mg/L group and up-regulated in the 50 mg/L, such as actin, alpha cardiac muscle 1 (P68033); actin, alpha skeletal muscle (P68134); actin, aortic smooth muscle (P62737); and actin, gamma-enteric smooth muscle (P63268). This up-regulation of actin filament-related proteins is often found in reactive astrocytes and may indicate astrocyte reactivity to an injury triggered by fluoride.

Another set of proteins directly associated with the cytoskeleton is the microtubule associated protein (MAP) group that act as stabilisers of microtubules^52^. The proteomic approach revealed the up-regulation of MAP 2 (P20357) and the exclusive expression of MAP 1A and 6 (Q9QYR6 and Q7TSJ2, respectively) in the 10 mg/L group compared with the control group. Q9QYR6 was found exclusively in 50 mg/L group compared with the control group but was down-regulated compared with the 10 mg/L group. In neurons, the MAP group is important to maintain synaptic plasticity^53^, along with other proteins such as postsynaptic density proteins 93 and 95, also known as disk large homolog 2 (Q91XM9) and 4 (Q62108), respectively, and synaptophysin (Q62277). These proteins compose the pre- and post-synaptic platforms and synaptic vesicle^54–56^). The 10 mg/L group showed exclusive regulation of Q91XM9 and Q62108 and down-regulation of Q62277 compared with the control group. This profile suggests that the lower fluoride concentration triggered an increase in synaptic activity, and the higher fluoride concentration led to a reduced synaptic activity, which may explain the behavioural results.

Following this perspective, exposure to 10 mg/L of fluoride caused down-regulation of calcium/calmodulin-dependent protein kinase type II subunits alpha (P11798) and beta (P28652) as well as calmodulin-1, 2 and 3 (P0DP26, P0DP27 and P0DP28, respectively), while exposure to 50 mg/L of fluoride caused up-regulation of these proteins. This protein complex plays several biological roles involving calcium signalling pathways, including synaptic plasticity^57^. It is known as one of the major sets of proteins present in post-synaptic platform and is involved in memory and learning^58,59^.

Based on the findings of this study, prolonged exposure to fluoride seems to be associated with an intense modulation of proteins related to synaptic transmission, changes that could underlie the functional impairments observed in the behavioural assessment and the molecular and morphological features. At 10 mg/L of fluoride, there was increased regulation of some synaptosomal components and an opposite pattern when exposed to 50 mg/L of fluoride. Besides, the neurodegeneration found in hippocampal regions is also indicative of how prolonged exposure to high concentrations of fluoride may trigger cognitive damage.

## Conclusions

In conclusion, prolonged exposure to the optimum fluoride level of artificially fluoridated water was not associated with cognitive impairments, while a higher concentration associated with fluorosis triggered memory and learning deficits, associated with a neuronal density reduction, suggestive of a neurodegenerative pattern in the hippocampus. Our results suggest additional investigations are needed with longer exposure times and at different ages to better elucidate whether the molecular alterations found at 10 mg/L are really harmless to cognitive function.

## Supplementary Information

Supplementary Tables.

## Data Availability

The quantitative and qualitative data used to support the findings of this study are included within the article or supporting information.
