# Supplementary material for: Prolonged exposure to high fluoride levels during adolescence to adulthood elicits molecular, morphological, and functional impairments in the hippocampus
Source: Sci Rep. 2023 Jul 8;13:11083. doi: 10.1038/s41598-023-38096-8 (PMC10329641; doi:10.1038/s41598-023-38096-8)
Supplement: Supplementary file 1 — Supplementary Tables. [file 41598_2023_38096_MOESM1_ESM.docx]

**SUPPLEMENTARY DATA**

**Article**: Prolonged exposure to high fluoride levels during adolescence to adulthood elicits molecular, morphological and functional impairments in the hippocampus

**Authors**: Leonardo Oliveira Bittencourt, Aline Dionizio, Maria Karolina Martins Ferreira, Walessa Alana Bragança Aragão, Sabrina de Carvalho Cartágenes, Bruna Puty, Cristiane do Socorro Ferraz Maia, Fatemeh Vida Zohoori, Marília Afonso Rabelo Buzalaf, Rafael Rodrigues Lima.

***Corresponding author:**

Rafael Rodrigues Lima, PhD

Laboratory of Functional and Structural Biology, Nº 125, Institute of Biological Sciences, Federal University of Pará; Augusto Corrêa street n. 01, Guamá, Belém-Pará 66075-110, Brazil

E-mail: [rafalima@ufpa.br](mailto:rafalima@ufpa.br)

**Supplementary table 1.** Complete list of biological processes according to Gene Ontology of the proteins found modulated between group comparisons.

| Group Comparison | Biological Process | Number of genes (%) |
| --- | --- | --- |
|  |  |  |
| 10mg/L vs. Control | Axon guidance | 13.92 |
|  | Regulation of axonogenesis | 12.66 |
|  | Dendritic spine morphogenesis | 8.86 |
|  | Mitochondrial ATP synthesis coupled proton transport | 7.59 |
|  | Glycolytic process through fructose-6-phosphate | 6.33 |
|  | Barbed-end actin filament capping | 6.33 |
|  | Hypothalamus gonadotrophin-releasing hormone neuron development | 5.06 |
|  | Central nervous system neuron axonogenesis | 3.80 |
|  | Pyruvate dehydrogenase (NAD+) activity | 3.80 |
|  | Positive regulation of ryanodine-sensitive calcium-release channel activity | 3.80 |
|  | Pyruvate kinase activity | 2.53 |
|  | Positive regulation of dendrite development | 2.53 |
|  | Axon target recognition | 2.53 |
|  | Motor neuron axon guidance | 2.53 |
|  | Positive regulation of natural killer cell differentiation | 2.53 |
|  | Positive regulation of histone acetylation | 2.53 |
|  | Negative regulation of histone acetylation | 2.53 |
|  | Positive regulation of JUN kinase activity | 2.53 |
|  | Negative regulation of JUN kinase activity | 2.53 |
|  | Activation of cysteine-type endopeptidase activity involved in apoptotic process by cytochrome c | 2.53 |
|  | Positive regulation of P-type sodium:potassium-exchanging transporter activity | 2.53 |
| 50 mg/L vs. Control | Axon guidance | 13.5 |
|  | Regulation of axonogenesis | 10.6 |
|  | Mitochondrial ATP synthesis coupled proton transport | 6.7 |
|  | Regulation of dendritic spine development | 6.7 |
|  | Transcription corepressor activity | 5.8 |
|  | Arp2/3 complex-mediated actin nucleation | 4.8 |
|  | Glycolytic process through fructose-6-phosphate | 4.8 |
|  | Ryanodine-sensitive calcium-release channel activity | 4.8 |
|  | Hypothalamus gonadotrophin-releasing hormone neuron development | 3.8 |
|  | Negative regulation of mRNA splicing, via spliceosome | 2.9 |
|  | Negative regulation of axonogenesis | 2.9 |
|  | Motor neuron axon guidance | 2.9 |
|  | Central nervous system neuron axonogenesis | 2.9 |
|  | Regulation of alternative mRNA splicing, via spliceosome | 1.9 |
|  | Retinal cone cell development | 1.9 |
|  | Collateral sprouting | 1.9 |
|  | Positive regulation of transcription initiation from RNA polymerase II promoter | 1.9 |
|  | Positive regulation of dendrite development | 1.9 |
|  | Nuclear receptor coactivator activity | 1.9 |
|  | Regulation of axon diameter | 1.9 |
|  | Regulation of isotype switching | 1.9 |
|  | cAMP-dependent protein kinase inhibitor activity | 1.9 |
|  | Regulation of barbed-end actin filament capping | 1.9 |
|  | High-affinity glutamate transmembrane transporter activity | 1.9 |
|  | Positive regulation of P-type sodium:potassium-exchanging transporter activity | 1.9 |
|  | Negative regulation of histone acetylation | 1.9 |
|  | Calcium-dependent activation of synaptic vesicle fusion | 1.9 |
| 50 mg/L vs. 10 mg/L | Regulation of axonogenesis | 12.6 |
|  | Axon guidance | 9.5 |
|  | Positive regulation of dendritic spine development | 9.5 |
|  | Arp2/3 complex-mediated actin nucleation | 7.4 |
|  | Mitochondrial ATP synthesis coupled proton transport | 6.3 |
|  | Glycolytic process through fructose-6-phosphate | 5.3 |
|  | Negative regulation of mRNA splicing, via spliceosome | 4.2 |
|  | Hypothalamus gonadotrophin-releasing hormone neuron development | 4.2 |
|  | Acetyl-CoA biosynthetic process from pyruvate | 4.2 |
|  | Pyruvate dehydrogenase (NAD+) activity | 4.2 |
|  | Retinal cone cell development | 3.2 |
|  | Regulation of alternative mRNA splicing, via spliceosome | 2.1 |
|  | Negative regulation of dendrite morphogenesis | 2.1 |
|  | Pyruvate kinase activity | 2.1 |
|  | Positive regulation of ATP biosynthetic process | 2.1 |
|  | Regulation of axon diameter | 2.1 |
|  | Histone H3-K14 acetylation | 2.1 |
|  | cAMP-dependent protein kinase inhibitor activity | 2.1 |
|  | Central nervous system projection neuron axonogenesis | 2.1 |
|  | Regulation of isotype switching | 2.1 |
|  | High-affinity glutamate transmembrane transporter activity | 2.1 |
|  | Positive regulation of P-type sodium:potassium-exchanging transporter activity | 2.1 |
|  | Calcium-dependent activation of synaptic vesicle fusion | 2.1 |
|  | Positive regulation of barbed-end actin filament capping | 2.1 |
|  | Auditory receptor cell morphogenesis | 2.1 |

**Supplementary table 2.** Identified proteins with expression significantly altered in the hippocampus of mice in 10 mg/L vs. control group.

| Accession Idª | Protein description | PLGS Score | Fold Change |
| --- | --- | --- | --- |
| Q64467 | Glyceraldehyde-3-phosphate dehydrogenase, testis-specific | 103.28 | 6.753 |
| Q9CQN1 | Heat shock protein 75 kDa, mitochondrial | 242.62 | 3.353 |
| Q8BZQ7 | Anaphase-promoting complex subunit 2 | 49.72 | 2.974 |
| P15626 | Glutathione S-transferase Mu 2 | 97.07 | 2.718 |
| Q80W21 | Glutathione S-transferase Mu 7 | 97.07 | 2.718 |
| Q9DB05 | Alpha-soluble NSF attachment protein | 54.03 | 2.586 |
| P29387 | Guanine nucleotide-binding protein subunit beta-4 | 984.78 | 2.411 |
| P62806 | Histone H4 | 1669.76 | 2.411 |
| Q61011 | Guanine nucleotide-binding protein G(I)/G(S)/G(T) subunit beta-3 | 150.23 | 2.363 |
| Q8CGP0 | Histone H2B type 3-B | 852.51 | 2.270 |
| Q8CGP2 | Histone H2B type 1-P | 1013.8 | 2.248 |
| Q64524 | Histone H2B type 2-E | 852.51 | 2.248 |
| Q6ZWY9 | Histone H2B type 1-C/E/G | 1013.8 | 2.226 |
| Q8CGP1 | Histone H2B type 1-K | 1013.8 | 2.226 |
| P10854 | Histone H2B type 1-M | 1013.8 | 2.226 |
| Q9D2U9 | Histone H2B type 3-A | 852.51 | 2.226 |
| Q64475 | Histone H2B type 1-B | 1013.8 | 2.203 |
| P10853 | Histone H2B type 1-F/J/L | 1013.8 | 2.203 |
| Q64478 | Histone H2B type 1-H | 1013.8 | 2.203 |
| Q64525 | Histone H2B type 2-B | 1013.8 | 2.203 |
| Q6ZPV2 | Chromatin-remodeling ATPase INO80 | 39.29 | 2.034 |
| P70296 | Phosphatidylethanolamine-binding protein 1 | 944.72 | 1.974 |
| Q91ZZ3 | Beta-synuclein | 76.97 | 1.954 |
| P28663 | Beta-soluble NSF attachment protein | 133.71 | 1.916 |
| Q3V132 | ADP/ATP translocase 4 | 49.88 | 1.859 |
| Q9JJZ2 | Tubulin alpha-8 chain | 4374.56 | 1.840 |
| O55042 | Alpha-synuclein | 477.8 | 1.786 |
| O08749 | Dihydrolipoyl dehydrogenase, mitochondrial | 94.03 | 1.786 |
| P14094 | Sodium/potassium-transporting ATPase subunit beta-1 | 604.37 | 1.786 |
| Q3UX10 | Tubulin alpha chain-like 3 | 517.77 | 1.751 |
| P48962 | ADP/ATP translocase 1 | 173.37 | 1.716 |
| C0HKE5 | Histone H2A type 1-G | 1343.38 | 1.682 |
| C0HKE8 | Histone H2A type 1-O | 1343.38 | 1.682 |
| Q6GSS7 | Histone H2A type 2-A | 1343.38 | 1.682 |
| C0HKE3 | Histone H2A type 1-D | 1343.38 | 1.665 |
| C0HKE4 | Histone H2A type 1-E | 1343.38 | 1.665 |
| C0HKE9 | Histone H2A type 1-P | 1343.38 | 1.665 |
| C0HKE1 | Histone H2A type 1-B | 1343.38 | 1.649 |
| Q8CGP5 | Histone H2A type 1-F | 1343.38 | 1.649 |
| C0HKE6 | Histone H2A type 1-I | 1343.38 | 1.649 |
| C0HKE7 | Histone H2A type 1-N | 1343.38 | 1.649 |
| Q64523 | Histone H2A type 2-C | 1343.38 | 1.649 |
| Q8BFU2 | Histone H2A type 3 | 1343.38 | 1.649 |
| O70456 | 14-3-3 protein sigma | 3068.34 | 1.632 |
| Q8CGP6 | Histone H2A type 1-H | 1343.38 | 1.632 |
| Q8R1M2 | Histone H2A.J | 1343.38 | 1.632 |
| C0HKE2 | Histone H2A type 1-C | 1343.38 | 1.616 |
| Q8CGP7 | Histone H2A type 1-K | 1343.38 | 1.600 |
| P39053 | Dynamin-1 | 4943.16 | 1.584 |
| Q8BZ98 | Dynamin-3 | 81.67 | 1.568 |
| P13595 | Neural cell adhesion molecule 1 | 40.83 | 1.553 |
| P46096 | Synaptotagmin-1 | 213.16 | 1.553 |
| Q9CZU6 | Citrate synthase, mitochondrial | 136.89 | 1.537 |
| P16125 | L-lactate dehydrogenase B chain | 607.14 | 1.537 |
| P62880 | Guanine nucleotide-binding protein G(I)/G(S)/G(T) subunit beta-2 | 1227.2 | 1.522 |
| P63328 | Serine/threonine-protein phosphatase 2B catalytic subunit alpha isoform | 209.5 | 1.507 |
| Q9WUA3 | ATP-dependent 6-phosphofructokinase, platelet type | 102.29 | 1.492 |
| P26443 | Glutamate dehydrogenase 1, mitochondrial | 717.95 | 1.492 |
| P61982 | 14-3-3 protein gamma | 3176.03 | 1.477 |
| P47857 | ATP-dependent 6-phosphofructokinase, muscle type | 66.63 | 1.477 |
| P20029 | Endoplasmic reticulum chaperone BiP | 441.99 | 1.477 |
| Q9D051 | Pyruvate dehydrogenase E1 component subunit beta, mitochondrial | 63.62 | 1.477 |
| O55131 | Septin-7 | 270.57 | 1.477 |
| P06745 | Glucose-6-phosphate isomerase | 613.13 | 1.462 |
| P05201 | Aspartate aminotransferase, cytoplasmic | 401.64 | 1.448 |
| Q9Z0E0 | Neurochondrin | 107.25 | 1.433 |
| P48453 | Serine/threonine-protein phosphatase 2B catalytic subunit beta isoform | 91.18 | 1.433 |
| P39054 | Dynamin-2 | 80.91 | 1.419 |
| Q9R0P9 | Ubiquitin carboxyl-terminal hydrolase isozyme L1 | 472.01 | 1.419 |
| P62874 | Guanine nucleotide-binding protein G(I)/G(S)/G(T) subunit beta-1 | 996.97 | 1.405 |
| P10649 | Glutathione S-transferase Mu 1 | 218.9 | 1.391 |
| P17427 | AP-2 complex subunit alpha-2 | 212.44 | 1.363 |
| P62631 | Elongation factor 1-alpha 2 | 167.42 | 1.363 |
| P43006 | Excitatory amino acid transporter 2 | 262.57 | 1.363 |
| P50396 | Rab GDP dissociation inhibitor alpha | 386.95 | 1.363 |
| Q60932 | Voltage-dependent anion-selective channel protein 1 | 1508.65 | 1.363 |
| P20357 | Microtubule-associated protein 2 | 267.4 | 1.350 |
| P09411 | Phosphoglycerate kinase 1 | 194.88 | 1.350 |
| Q9DD03 | Ras-related protein Rab-13 | 637.26 | 1.350 |
| P08228 | Superoxide dismutase [Cu-Zn] | 521.38 | 1.350 |
| P00342 | L-lactate dehydrogenase C chain | 515.53 | 1.336 |
| P62259 | 14-3-3 protein epsilon | 2983.16 | 1.323 |
| Q6PIE5 | Sodium/potassium-transporting ATPase subunit alpha-2 | 1008.51 | 1.323 |
| P00405 | Cytochrome c oxidase subunit 2 | 331.92 | 1.310 |
| Q61598 | Rab GDP dissociation inhibitor beta | 449.42 | 1.310 |
| Q60930 | Voltage-dependent anion-selective channel protein 2 | 862.66 | 1.310 |
| Q6PIC6 | Sodium/potassium-transporting ATPase subunit alpha-3 | 1247.9 | 1.297 |
| Q9DB20 | ATP synthase subunit O, mitochondrial | 1284.26 | 1.284 |
| P11499 | Heat shock protein HSP 90-beta | 347.11 | 1.284 |
| P06151 | L-lactate dehydrogenase A chain | 718.83 | 1.284 |
| Q9CZT8 | Ras-related protein Rab-3B | 641.75 | 1.284 |
| Q9D0M3 | Cytochrome c1, heme protein, mitochondrial | 154.1 | 1.271 |
| Q01768 | Nucleoside diphosphate kinase B | 330.98 | 1.271 |
| Q8VEM8 | Phosphate carrier protein, mitochondrial | 303.54 | 1.271 |
| P62821 | Ras-related protein Rab-1A | 1356.49 | 1.271 |
| P35276 | Ras-related protein Rab-3D | 697.93 | 1.271 |
| P46460 | Vesicle-fusing ATPase | 225.46 | 1.271 |
| Q9CZ13 | Cytochrome b-c1 complex subunit 1, mitochondrial | 232.11 | 1.259 |
| P63011 | Ras-related protein Rab-3A | 1170.72 | 1.259 |
| P62823 | Ras-related protein Rab-3C | 641.75 | 1.259 |
| P55258 | Ras-related protein Rab-8A | 1356.49 | 1.259 |
| Q9CQV8 | 14-3-3 protein beta/alpha | 3251.5 | 1.246 |
| P30275 | Creatine kinase U-type, mitochondrial | 521.45 | 1.246 |
| P12787 | Cytochrome c oxidase subunit 5A, mitochondrial | 1359.6 | 1.246 |
| P61027 | Ras-related protein Rab-10 | 1356.49 | 1.246 |
| Q8K386 | Ras-related protein Rab-15 | 1273.06 | 1.246 |
| P61028 | Ras-related protein Rab-8B | 1356.49 | 1.246 |
| P62814 | V-type proton ATPase subunit B, brain isoform | 95.93 | 1.246 |
| Q9D1G1 | Ras-related protein Rab-1B | 1356.49 | 1.234 |
| Q504M8 | Ras-related protein Rab-26 | 1356.49 | 1.234 |
| Q6PHN9 | Ras-related protein Rab-35 | 1356.49 | 1.234 |
| Q8CG50 | Ras-related protein Rab-43 | 1356.49 | 1.234 |
| O35963 | Ras-related protein Rab-33B | 1196.69 | 1.221 |
| P63101 | 14-3-3 protein zeta/delta | 4286.07 | 1.209 |
| Q923T9 | Calcium/calmodulin-dependent protein kinase type II subunit gamma | 1360.71 | 1.209 |
| Q61171 | Peroxiredoxin-2 | 770.66 | 1.209 |
| Q923S9 | Ras-related protein Rab-30 | 1356.49 | 1.209 |
| Q8BHD0 | Ras-related protein Rab-39A | 1310.98 | 1.209 |
| P01831 | Thy-1 membrane glycoprotein | 202.4 | 1.209 |
| P02089 | Hemoglobin subunit beta-2 | 1971.96 | 1.197 |
| P16546 | Spectrin alpha chain, non-erythrocytic 1 | 133.67 | 1.197 |
| P63038 | 60 kDa heat shock protein, mitochondrial | 125.91 | 1.174 |
| P19783 | Cytochrome c oxidase subunit 4 isoform 1, mitochondrial | 321.26 | 1.174 |
| P15532 | Nucleoside diphosphate kinase A | 330.98 | 1.174 |
| Q62261 | Spectrin beta chain, non-erythrocytic 1 | 118.82 | 1.150 |
| P97427 | Dihydropyrimidinase-related protein 1 | 1567.8 | 1.094 |
| Q61595 | Kinectin | 239.12 | -0.167 |
| O70167 | Phosphatidylinositol 4-phosphate 3-kinase C2 domain-containing subunit gamma | 116.13 | -0.228 |
| P02088 | Hemoglobin subunit beta-1 | 3543.4 | -0.323 |
| P01942 | Hemoglobin subunit alpha | 2208.07 | -0.343 |
| P02104 | Hemoglobin subunit epsilon-Y2 | 1971.96 | -0.353 |
| P61164 | Alpha-centractin | 251.17 | -0.387 |
| P68372 | Tubulin beta-4B chain | 28843.51 | -0.387 |
| P99024 | Tubulin beta-5 chain | 35888.25 | -0.399 |
| P68134 | Actin, alpha skeletal muscle | 24717.44 | -0.403 |
| P60202 | Myelin proteolipid protein | 5128.56 | -0.403 |
| Q9ERD7 | Tubulin beta-3 chain | 25756.16 | -0.407 |
| P68373 | Tubulin alpha-1C chain | 11309.22 | -0.411 |
| Q7TMM9 | Tubulin beta-2A chain | 37520.65 | -0.411 |
| P62737 | Actin, aortic smooth muscle | 24551.85 | -0.415 |
| P68369 | Tubulin alpha-1A chain | 12359.69 | -0.415 |
| P05064 | Fructose-bisphosphate aldolase A | 3093.45 | -0.419 |
| Q8R5C5 | Beta-centractin | 251.17 | -0.423 |
| P05214 | Tubulin alpha-3 chain | 7666.05 | -0.423 |
| O08553 | Dihydropyrimidinase-related protein 2 | 6913.4 | -0.427 |
| Q9DC51 | Guanine nucleotide-binding protein G(i) subunit alpha | 1623.52 | -0.427 |
| B2RSH2 | Guanine nucleotide-binding protein G(i) subunit alpha-1 | 1623.52 | -0.432 |
| P08752 | Guanine nucleotide-binding protein G(i) subunit alpha-2 | 1623.52 | -0.432 |
| Q8CGK7 | Guanine nucleotide-binding protein G(olf) subunit alpha | 1628.32 | -0.432 |
| P17156 | Heat shock-related 70 kDa protein 2 | 933.05 | -0.463 |
| P16330 | 2',3'-cyclic-nucleotide 3'-phosphodiesterase | 887.7 | -0.482 |
| P11798 | Calcium/calmodulin-dependent protein kinase type II subunit alpha | 4430.82 | -0.487 |
| P17879 | Heat shock 70 kDa protein 1B | 891.74 | -0.487 |
| P28652 | Calcium/calmodulin-dependent protein kinase type II subunit beta | 1850.3 | -0.492 |
| Q61696 | Heat shock 70 kDa protein 1A | 891.74 | -0.492 |
| P18872 | Guanine nucleotide-binding protein G(o) subunit alpha | 2986.96 | -0.527 |
| Q03265 | ATP synthase subunit alpha, mitochondrial | 2101.69 | -0.533 |
| Q62277 | Synaptophysin | 517.81 | -0.543 |
| P04370 | Myelin basic protein | 9022.31 | -0.549 |
| Q9Z1W8 | Potassium-transporting ATPase alpha chain 2 | 514.97 | -0.554 |
| Q9WV27 | Sodium/potassium-transporting ATPase subunit alpha-4 | 531.64 | -0.577 |
| P53657 | Pyruvate kinase PKLR | 382.02 | -0.583 |
| P10126 | Elongation factor 1-alpha 1 | 355.16 | -0.607 |
| P52480 | Pyruvate kinase PKM | 5072.32 | -0.613 |
| P08249 | Malate dehydrogenase, mitochondrial | 3024.44 | -0.625 |
| O08599 | Syntaxin-binding protein 1 | 1908.06 | -0.644 |
| Q68FD5 | Clathrin heavy chain 1 | 227.48 | -0.651 |
| Q9D6P8 | Calmodulin-like protein 3 | 89.23 | -0.664 |
| P05202 | Aspartate aminotransferase, mitochondrial | 883.71 | -0.719 |
| P08553 | Neurofilament medium polypeptide | 365.1 | -0.719 |
| P0DP27 | Calmodulin-2 | 1448.27 | -0.733 |
| P16627 | Heat shock 70 kDa protein 1-like | 864.62 | -0.733 |
| Q64332 | Synapsin-2 | 356.6 | -0.733 |
| P0DP26 | Calmodulin-1 | 1448.27 | -0.741 |
| P0DP28 | Calmodulin-3 | 1448.27 | -0.741 |
| P60879 | Synaptosomal-associated protein 25 | 197.72 | -0.748 |
| P62962 | Profilin-1 | 1570.27 | -0.756 |
| P46660 | Alpha-internexin | 52.44 | -0.779 |
| Q99PT1 | Rho GDP-dissociation inhibitor 1 | 335.24 | -0.819 |
| O88935 | Synapsin-1 | 752.24 | -0.827 |
| P17183 | Gamma-enolase | 3347.39 | -0.844 |
| P16858 | Glyceraldehyde-3-phosphate dehydrogenase | 13052.28 | -0.844 |
| P18760 | Cofilin-1 | 1063.08 | -0.852 |
| Q04447 | Creatine kinase B-type | 3079.53 | -0.869 |
| P17751 | Triosephosphate isomerase | 1422.35 | -0.869 |
| P68033 | Actin, alpha cardiac muscle 1 | 24755.44 | -0.878 |
| Q922F4 | Tubulin beta-6 chain | 14837.39 | -0.887 |
| P21550 | Beta-enolase | 1943.89 | -0.905 |
| Q8BFZ3 | Beta-actin-like protein 2 | 7162.73 | -0.923 |
| P68368 | Tubulin alpha-4A chain | 14150.28 | -0.923 |
| P63268 | Actin, gamma-enteric smooth muscle | 24558.29 | -0.932 |
| Q3TXS7 | 26S proteasome non-ATPase regulatory subunit 1 | 114.46 | Control |
| Q9R0Q6 | Actin-related protein 2/3 complex subunit 1A | 70.32 | Control |
| Q99JY9 | Actin-related protein 3 | 184.91 | Control |
| Q9QXN3 | Activating signal cointegrator 1 | 64.68 | Control |
| P40124 | Adenylyl cyclase-associated protein 1 | 340.87 | Control |
| E9Q394 | A-kinase anchor protein 13 | 49.13 | Control |
| Q9JII6 | Aldo-keto reductase family 1 member A1 | 122.11 | Control |
| P84091 | AP-2 complex subunit mu | 138.32 | Control |
| Q91V80 | Apolipoprotein F | 151.7 | Control |
| O88879 | Apoptotic protease-activating factor 1 | 85 | Control |
| P39654 | Arachidonate 15-lipoxygenase | 45.59 | Control |
| P98203 | Armadillo repeat protein deleted in velo-cardio-facial syndrome homolog | 51.81 | Control |
| Q9DBR3 | Armadillo repeat-containing protein 8 | 35.27 | Control |
| Q91YH5 | Atlastin-3 | 105.62 | Control |
| Q9CQQ7 | ATP synthase F(0) complex subunit B1, mitochondrial | 126.25 | Control |
| P03930 | ATP synthase protein 8 | 681.06 | Control |
| Q9D3D9 | ATP synthase subunit delta, mitochondrial | 366.15 | Control |
| E9PX95 | ATP-binding cassette sub-family A member 17 | 41.04 | Control |
| B5X0E4 | ATP-binding cassette sub-family B member 5 | 87.08 | Control |
| Q9DBI2 | Bardet-Biedl syndrome 10 protein homolog | 54.26 | Control |
| Q8CDE2 | Calicin | 49.27 | Control |
| Q5PR69 | Cancer-related regulator of actin dynamics | 43.25 | Control |
| Q9CTG6 | Cation-transporting ATPase 13A2 | 70.86 | Control |
| Q5PR68 | Centrosomal protein of 112 kDa | 47.55 | Control |
| Q3UPP8 | Centrosomal protein of 63 kDa | 35.86 | Control |
| Q9Z0X4 | cGMP-inhibited 3',5'-cyclic phosphodiesterase A | 25.5 | Control |
| Q61548 | Clathrin coat assembly protein AP180 | 76.8 | Control |
| Q8CDV0 | Coiled-coil domain-containing protein 178 | 42.91 | Control |
| Q2UY11 | Collagen alpha-1(XXVIII) chain | 102.74 | Control |
| Q6DFV1 | Condensin-2 complex subunit G2 | 221.95 | Control |
| Q6ZQ38 | Cullin-associated NEDD8-dissociated protein 1 | 46.13 | Control |
| Q62425 | Cytochrome c oxidase subunit NDUFA4 | 1346.01 | Control |
| Q8BPN8 | DmX-like protein 2 | 27.86 | Control |
| Q8BHK9 | DNA excision repair protein ERCC-6-like | 16.41 | Control |
| P33611 | DNA polymerase alpha subunit B | 77.5 | Control |
| P70388 | DNA repair protein RAD50 | 47.24 | Control |
| Q8C7R7 | DNA-binding protein RFX6 | 56.61 | Control |
| Q3V0Q1 | Dynein heavy chain 12, axonemal | 42.8 | Control |
| Q91XQ0 | Dynein heavy chain 8, axonemal | 23.7 | Control |
| Q6ZQM0 | E3 ubiquitin-protein ligase rififylin | 69.42 | Control |
| Q3TGW2 | Endonuclease/exonuclease/phosphatase family domain-containing protein 1 | 90.5 | Control |
| P08113 | Endoplasmin | 28.95 | Control |
| Q8R418 | Endoribonuclease Dicer | 45.65 | Control |
| Q3TDN2 | FAS-associated factor 2 | 115.28 | Control |
| Q922J9 | Fatty acyl-CoA reductase 1 | 64.67 | Control |
| Q80ZA4 | Fibrocystin-L | 23.87 | Control |
| Q8CI03 | FLYWCH-type zinc finger-containing protein 1 | 142.23 | Control |
| P49772 | Fms-related tyrosine kinase 3 ligand | 100.11 | Control |
| Q8K0C9 | GDP-mannose 4,6 dehydratase | 51.77 | Control |
| P97324 | Glucose-6-phosphate 1-dehydrogenase 2 | 38.33 | Control |
| Q9D4P7 | Glutathione S-transferase theta-4 | 127.3 | Control |
| Q8CI94 | Glycogen phosphorylase, brain form | 54.74 | Control |
| Q9ET01 | Glycogen phosphorylase, liver form | 49.53 | Control |
| Q9WUB3 | Glycogen phosphorylase, muscle form | 33.78 | Control |
| Q3UFB7 | High affinity nerve growth factor receptor | 46.22 | Control |
| Q8C2B3 | Histone deacetylase 7 | 46.29 | Control |
| Q63ZW7 | InaD-like protein | 108.24 | Control |
| P56477 | Interferon regulatory factor 5 | 136.19 | Control |
| Q6IME9 | Keratin, type II cytoskeletal 72 | 52.82 | Control |
| P28738 | Kinesin heavy chain isoform 5C | 49.49 | Control |
| Q9QXL2 | Kinesin-like protein KIF21A | 70.96 | Control |
| Q3URE9 | Leucine-rich repeat and immunoglobulin-like domain-containing nogo receptor-interacting protein 2 | 106.78 | Control |
| Q80U28 | MAP kinase-activating death domain protein | 82.2 | Control |
| P53349 | Mitogen-activated protein kinase kinase kinase 1 | 42.29 | Control |
| Q99LC3 | NADH dehydrogenase [ubiquinone] 1 alpha subcomplex subunit 10, mitochondrial | 590.31 | Control |
| Q9D6J5 | NADH dehydrogenase [ubiquinone] 1 beta subcomplex subunit 8, mitochondrial | 74.34 | Control |
| Q810U3 | Neurofascin | 81.34 | Control |
| Q8CH77 | Neuron navigator 1 | 37.69 | Control |
| Q6DFV7 | Nuclear receptor coactivator 7 | 55.17 | Control |
| Q8K4K6 | Pantothenate kinase 1 | 56.14 | Control |
| Q8R1G6 | PDZ and LIM domain protein 2 | 54.81 | Control |
| Q8CGT6 | Piwi-like protein 4 | 77.12 | Control |
| Q7TQ62 | Podocan | 87.39 | Control |
| Q91Z31 | Polypyrimidine tract-binding protein 2 | 53.52 | Control |
| Q99MQ1 | Protein bicaudal C homolog 1 | 60.29 | Control |
| P27773 | Protein disulfide-isomerase A3 | 57.17 | Control |
| Q9DAI6 | Protein FAM135B | 46.17 | Control |
| E9Q8I9 | Protein furry homolog | 49.48 | Control |
| Q61644 | Protein kinase C and casein kinase substrate in neurons protein 1 | 140.75 | Control |
| Q8R1F1 | Protein Niban 2 | 87.89 | Control |
| E5FYH1 | Protein TOPAZ1 | 48.39 | Control |
| Q8K3V4 | Protein-arginine deiminase type-6 | 54.78 | Control |
| Q91Y02 | Protocadherin beta-18 | 65 | Control |
| P35486 | Pyruvate dehydrogenase E1 component subunit alpha, somatic form, mitochondrial | 75.76 | Control |
| Q8VCD6 | Receptor expression-enhancing protein 2 | 103.82 | Control |
| Q62132 | Receptor-type tyrosine-protein phosphatase R | 43.05 | Control |
| B9EKR1 | Receptor-type tyrosine-protein phosphatase zeta | 21.41 | Control |
| Q8K0T0 | Reticulon-1 | 68.24 | Control |
| Q9ES97 | Reticulon-3 | 69.89 | Control |
| Q80U35 | Rho guanine nucleotide exchange factor 17 | 48.63 | Control |
| Q9D3G9 | Rho-related GTP-binding protein RhoH | 86.75 | Control |
| Q3UZ01 | RNA-binding region-containing protein 3 | 100.41 | Control |
| P60330 | Separin | 62.04 | Control |
| Q91ZR4 | Serine/threonine-protein kinase Nek8 | 39.5 | Control |
| Q9JK88 | Serpin I2 | 57.22 | Control |
| Q60665 | Ski-like protein | 41.91 | Control |
| Q8K596 | Sodium/calcium exchanger 2 | 64.72 | Control |
| Q8K078 | Solute carrier organic anion transporter family member 4A1 | 58.66 | Control |
| Q9D3S3 | Sorting nexin-29 | 26.68 | Control |
| Q5U4C3 | Splicing factor, arginine/serine-rich 19 | 29.48 | Control |
| F6XZJ7 | Sterile alpha motif domain-containing protein 15 | 44.9 | Control |
| P83093 | Stromal interaction molecule 2 | 48.68 | Control |
| Q9WUM5 | Succinate--CoA ligase [ADP/GDP-forming] subunit alpha, mitochondrial | 340.57 | Control |
| Q9Z2I9 | Succinate--CoA ligase [ADP-forming] subunit beta, mitochondrial | 48.27 | Control |
| O55100 | Synaptogyrin-1 | 262.69 | Control |
| Q8CHC4 | Synaptojanin-1 | 95.51 | Control |
| P80316 | T-complex protein 1 subunit epsilon | 200.72 | Control |
| P42932 | T-complex protein 1 subunit theta | 149.24 | Control |
| Q80W22 | Threonine synthase-like 2 | 96.5 | Control |
| Q9Z1T2 | Thrombospondin-4 | 52.59 | Control |
| Q61286 | Transcription factor 12 | 98.9 | Control |
| P42669 | Transcriptional activator protein Pur-alpha | 126.95 | Control |
| Q9R1Q8 | Transgelin-3 | 138.09 | Control |
| Q7TQI3 | Ubiquitin thioesterase OTUB1 | 99.66 | Control |
| Q9D906 | Ubiquitin-like modifier-activating enzyme ATG7 | 56.75 | Control |
| P40336 | Vacuolar protein sorting-associated protein 26A | 139.42 | Control |
| P29788 | Vitronectin | 55.89 | Control |
| Q9Z1B3 | 1-phosphatidylinositol 4,5-bisphosphate phosphodiesterase beta-1 | 41.48 | 10 mg/L |
| Q60597 | 2-oxoglutarate dehydrogenase, mitochondrial | 92.99 | 10 mg/L |
| P61922 | 4-aminobutyrate aminotransferase, mitochondrial | 573.78 | 10 mg/L |
| P10852 | 4F2 cell-surface antigen heavy chain | 132.09 | 10 mg/L |
| Q5SSL4 | Active breakpoint cluster region-related protein | 51.4 | 10 mg/L |
| P31786 | Acyl-CoA-binding protein | 1471.59 | 10 mg/L |
| P84078 | ADP-ribosylation factor 1 | 1615.26 | 10 mg/L |
| Q8BSL7 | ADP-ribosylation factor 2 | 1134.59 | 10 mg/L |
| P61205 | ADP-ribosylation factor 3 | 1615.26 | 10 mg/L |
| P61750 | ADP-ribosylation factor 4 | 815.33 | 10 mg/L |
| P84084 | ADP-ribosylation factor 5 | 815.33 | 10 mg/L |
| Q80Y20 | Alkylated DNA repair protein alkB homolog 8 | 129.16 | 10 mg/L |
| Q9QYC0 | Alpha-adducin | 480.63 | 10 mg/L |
| Q7TQF7 | Amphiphysin | 81.33 | 10 mg/L |
| Q8C6Y6 | Ankyrin repeat and SOCS box protein 14 | 57.7 | 10 mg/L |
| O35643 | AP-1 complex subunit beta-1 | 60.21 | 10 mg/L |
| Q9DBG3 | AP-2 complex subunit beta | 110.45 | 10 mg/L |
| P06728 | Apolipoprotein A-IV | 180.65 | 10 mg/L |
| Q99KN1 | Arrestin domain-containing protein 1 | 175.26 | 10 mg/L |
| Q9DCX2 | ATP synthase subunit d, mitochondrial | 407.79 | 10 mg/L |
| P97450 | ATP synthase-coupling factor 6, mitochondrial | 785.56 | 10 mg/L |
| Q9DC29 | ATP-binding cassette sub-family B member 6, mitochondrial | 32.46 | 10 mg/L |
| Q99PU8 | ATP-dependent RNA helicase DHX30 | 149.18 | 10 mg/L |
| Q80XK6 | Autophagy-related protein 2 homolog B | 56.63 | 10 mg/L |
| Q9Z2H5 | Band 4.1-like protein 1 | 55.35 | 10 mg/L |
| Q9WV92 | Band 4.1-like protein 3 | 91.02 | 10 mg/L |
| Q9QYB8 | Beta-adducin | 84.36 | 10 mg/L |
| Q8BKX1 | Brain-specific angiogenesis inhibitor 1-associated protein 2 | 130.66 | 10 mg/L |
| Q61361 | Brevican core protein | 68.86 | 10 mg/L |
| Q9R1S8 | Calpain-7 | 68.21 | 10 mg/L |
| Q65CL1 | Catenin alpha-3 | 54.03 | 10 mg/L |
| P35762 | CD81 antigen | 627.76 | 10 mg/L |
| Q99N28 | Cell adhesion molecule 3 | 230.14 | 10 mg/L |
| Q6A065 | Centrosomal protein of 170 kDa | 88.12 | 10 mg/L |
| Q9EPU4 | Cleavage and polyadenylation specificity factor subunit 1 | 137.64 | 10 mg/L |
| Q8CDI7 | Coiled-coil domain-containing protein 150 | 73.6 | 10 mg/L |
| P63040 | Complexin-1 | 31.93 | 10 mg/L |
| P84086 | Complexin-2 | 429.62 | 10 mg/L |
| Q8CI04 | Conserved oligomeric Golgi complex subunit 3 | 29.61 | 10 mg/L |
| Q9DB77 | Cytochrome b-c1 complex subunit 2, mitochondrial | 430.39 | 10 mg/L |
| P19536 | Cytochrome c oxidase subunit 5B, mitochondrial | 392.36 | 10 mg/L |
| P56391 | Cytochrome c oxidase subunit 6B1 | 192.35 | 10 mg/L |
| P48771 | Cytochrome c oxidase subunit 7A2, mitochondrial | 514.4 | 10 mg/L |
| P62897 | Cytochrome c, somatic | 498.73 | 10 mg/L |
| Q8C4S8 | DENN domain-containing protein 2A | 64.83 | 10 mg/L |
| Q9EQF6 | Dihydropyrimidinase-related protein 5 | 70.73 | 10 mg/L |
| Q811D0 | Disks large homolog 1 | 78.4 | 10 mg/L |
| Q91XM9 | Disks large homolog 2 | 98.04 | 10 mg/L |
| Q62108 | Disks large homolog 4 | 206.56 | 10 mg/L |
| Q6PFD5 | Disks large-associated protein 3 | 52.24 | 10 mg/L |
| Q3UYV8 | Dynein assembly factor 3, axonemal | 78.6 | 10 mg/L |
| Q8BFR5 | Elongation factor Tu, mitochondrial | 111.39 | 10 mg/L |
| Q9DCS3 | Enoyl-[acyl-carrier-protein] reductase, mitochondrial | 119.84 | 10 mg/L |
| Q0VAV2 | Exophilin-5 | 38.17 | 10 mg/L |
| Q9ERK4 | Exportin-2 | 81.71 | 10 mg/L |
| P47754 | F-actin-capping protein subunit alpha-2 | 227.89 | 10 mg/L |
| Q80X90 | Filamin-B | 32.25 | 10 mg/L |
| P97807 | Fumarate hydratase, mitochondrial | 153.96 | 10 mg/L |
| D3Z7P3 | Glutaminase kidney isoform, mitochondrial | 99.06 | 10 mg/L |
| P19157 | Glutathione S-transferase P 1 | 1099.34 | 10 mg/L |
| P46425 | Glutathione S-transferase P 2 | 495.07 | 10 mg/L |
| Q61316 | Heat shock 70 kDa protein 4 | 102.54 | 10 mg/L |
| P48722 | Heat shock 70 kDa protein 4L | 93.19 | 10 mg/L |
| Q61699 | Heat shock protein 105 kDa | 59.3 | 10 mg/L |
| Q9WV07 | Hydroperoxide isomerase ALOXE3 | 59.28 | 10 mg/L |
| Q3KNY0 | Immunoglobulin-like and fibronectin type III domain-containing protein 1 | 29.85 | 10 mg/L |
| Q571I4 | Inactive tyrosine-protein kinase PRAG1 | 58.94 | 10 mg/L |
| Q80V86 | Integrator complex subunit 8 | 71.24 | 10 mg/L |
| Q60625 | Intercellular adhesion molecule 5 | 48.95 | 10 mg/L |
| Q9D8C4 | Interferon-induced 35 kDa protein homolog | 307.48 | 10 mg/L |
| Q9QZ85 | Interferon-inducible GTPase 1 | 58.34 | 10 mg/L |
| Q62406 | Interleukin-1 receptor-associated kinase 1 | 142.9 | 10 mg/L |
| P11369 | LINE-1 retrotransposable element ORF2 protein | 40.09 | 10 mg/L |
| Q9JI18 | Low-density lipoprotein receptor-related protein 1B | 39.31 | 10 mg/L |
| Q9JJ78 | Lymphokine-activated killer T-cell-originated protein kinase | 69.55 | 10 mg/L |
| Q9QYR6 | Microtubule-associated protein 1A | 42.54 | 10 mg/L |
| Q7TSJ2 | Microtubule-associated protein 6 | 116.38 | 10 mg/L |
| Q9D6M3 | Mitochondrial glutamate carrier 1 | 1913.94 | 10 mg/L |
| Q9DB41 | Mitochondrial glutamate carrier 2 | 264.97 | 10 mg/L |
| Q63844 | Mitogen-activated protein kinase 3 | 130.87 | 10 mg/L |
| O08539 | Myc box-dependent-interacting protein 1 | 320.05 | 10 mg/L |
| Q61885 | Myelin-oligodendrocyte glycoprotein | 287.13 | 10 mg/L |
| Q9D6J6 | NADH dehydrogenase [ubiquinone] flavoprotein 2, mitochondrial | 554.17 | 10 mg/L |
| P52503 | NADH dehydrogenase [ubiquinone] iron-sulfur protein 6, mitochondrial | 194.46 | 10 mg/L |
| Q91VD9 | NADH-ubiquinone oxidoreductase 75 kDa subunit, mitochondrial | 303.78 | 10 mg/L |
| Q6GQX2 | Nck-associated protein 5-like | 147.69 | 10 mg/L |
| Q8R007 | Nectin-4 | 72.22 | 10 mg/L |
| P70211 | Netrin receptor DCC | 45.19 | 10 mg/L |
| P06837 | Neuromodulin | 347.57 | 10 mg/L |
| P35802 | Neuronal membrane glycoprotein M6-a | 67.63 | 10 mg/L |
| Q9QXX8 | Nuclear fragile X mental retardation-interacting protein 1 | 98.96 | 10 mg/L |
| Q61937 | Nucleophosmin | 82.67 | 10 mg/L |
| O54998 | Peptidyl-prolyl cis-trans isomerase FKBP7 | 99.86 | 10 mg/L |
| P35700 | Peroxiredoxin-1 | 796.59 | 10 mg/L |
| O08807 | Peroxiredoxin-4 | 70.37 | 10 mg/L |
| Q5BL07 | Peroxisome biogenesis factor 1 | 140.96 | 10 mg/L |
| Q91YL7 | PGAP2-interacting protein | 52.95 | 10 mg/L |
| O70250 | Phosphoglycerate mutase 2 | 112.61 | 10 mg/L |
| P98199 | Phospholipid-transporting ATPase ID | 48.24 | 10 mg/L |
| Q8CDG1 | Piwi-like protein 2 | 131.71 | 10 mg/L |
| P0CG49 | Polyubiquitin-B | 1318.22 | 10 mg/L |
| P0CG50 | Polyubiquitin-C | 1318.22 | 10 mg/L |
| P67778 | Prohibitin | 102.37 | 10 mg/L |
| O35129 | Prohibitin-2 | 402.09 | 10 mg/L |
| Q9QXV0 | ProSAAS | 113.13 | 10 mg/L |
| Q61207 | Prosaposin | 74.57 | 10 mg/L |
| O55125 | Protein NipSnap homolog 1 | 173.16 | 10 mg/L |
| Q9CU24 | Protein THEMIS3 | 47.42 | 10 mg/L |
| P35294 | Ras-related protein Rab-19 | 293.2 | 10 mg/L |
| P24549 | Retinal dehydrogenase 1 | 132.8 | 10 mg/L |
| Q8BRH3 | Rho GTPase-activating protein 19 | 82.39 | 10 mg/L |
| Q8BWA8 | Rho guanine nucleotide exchange factor 19 | 263.26 | 10 mg/L |
| Q9JLC8 | Sacsin | 79.77 | 10 mg/L |
| Q9CZC8 | Secernin-1 | 250.9 | 10 mg/L |
| Q8C650 | Septin-10 | 66.2 | 10 mg/L |
| Q8C1B7 | Septin-11 | 109.63 | 10 mg/L |
| Q9DA97 | Septin-14 | 66.2 | 10 mg/L |
| Q9R1T4 | Septin-6 | 291.67 | 10 mg/L |
| B2RXR6 | Serine/threonine-protein phosphatase 6 regulatory ankyrin repeat subunit B | 116.3 | 10 mg/L |
| Q4VA53 | Sister chromatid cohesion protein PDS5 homolog B | 44.45 | 10 mg/L |
| Q3UHA3 | Spatacsin | 34.61 | 10 mg/L |
| P52019 | Squalene monooxygenase | 139.92 | 10 mg/L |
| P61264 | Syntaxin-1B | 374.9 | 10 mg/L |
| Q71LX4 | Talin-2 | 48.84 | 10 mg/L |
| Q3UR70 | Transforming growth factor-beta receptor-associated protein 1 | 101.94 | 10 mg/L |
| Q00993 | Tyrosine-protein kinase receptor UFO | 51.64 | 10 mg/L |
| P56399 | Ubiquitin carboxyl-terminal hydrolase 5 | 68.64 | 10 mg/L |
| P62983 | Ubiquitin-40S ribosomal protein S27a | 1318.22 | 10 mg/L |
| P62984 | Ubiquitin-60S ribosomal protein L40 | 1318.22 | 10 mg/L |
| Q9DBP5 | UMP-CMP kinase | 91.98 | 10 mg/L |
| P63024 | Vesicle-associated membrane protein 3 | 40.24 | 10 mg/L |
| P62761 | Visinin-like protein 1 | 134.82 | 10 mg/L |
| Q60931 | Voltage-dependent anion-selective channel protein 3 | 102.28 | 10 mg/L |
| P51863 | V-type proton ATPase subunit d 1 | 159.48 | 10 mg/L |
| P50518 | V-type proton ATPase subunit E 1 | 302.13 | 10 mg/L |
| Q8BVE3 | V-type proton ATPase subunit H | 172.23 | 10 mg/L |

ªUniprot accession ID retrieved from uniport.org database; Negative values of fold change mean down-regulated proteins; 10 mg/L or Control in fold change column means that protein was exclusively found in the respective experimental.

**Supplementary table 3.** Identified proteins with expression significantly altered in the hippocampus of mice in 50 mg/L vs. control group.

| Accession Idª | Protein description | PLGS Score | Fold Change |
| --- | --- | --- | --- |
| Q8BZQ7 | Anaphase-promoting complex subunit 2 | 49.72 | 8.41 |
| Q3TGW2 | Endonuclease/exonuclease/phosphatase family domain-containing protein 1 | 90.5 | 4.18 |
| Q64467 | Glyceraldehyde-3-phosphate dehydrogenase, testis-specific | 103.28 | 2.69 |
| Q9DB05 | Alpha-soluble NSF attachment protein | 54.03 | 2.27 |
| P15626 | Glutathione S-transferase Mu 2 | 97.07 | 2.16 |
| A2AQ07 | Tubulin beta-1 chain | 527.66 | 2.08 |
| Q80W21 | Glutathione S-transferase Mu 7 | 97.07 | 2.03 |
| Q9Z1W8 | Potassium-transporting ATPase alpha chain 2 | 514.97 | 2.01 |
| Q64436 | Potassium-transporting ATPase alpha chain 1 | 110.77 | 1.97 |
| P02104 | Hemoglobin subunit epsilon-Y2 | 1972 | 1.88 |
| P26883 | Peptidyl-prolyl cis-trans isomerase FKBP1A | 1709.6 | 1.88 |
| P13595 | Neural cell adhesion molecule 1 | 40.83 | 1.84 |
| P09041 | Phosphoglycerate kinase 2 | 133.46 | 1.84 |
| Q8BFZ3 | Beta-actin-like protein 2 | 7162.7 | 1.80 |
| P48774 | Glutathione S-transferase Mu 5 | 47.83 | 1.79 |
| P0DP26 | Calmodulin-1 | 1448.3 | 1.77 |
| Q7TMM9 | Tubulin beta-2A chain | 37521 | 1.77 |
| O55131 | Septin-7 | 270.57 | 1.73 |
| Q922F4 | Tubulin beta-6 chain | 14837 | 1.73 |
| Q9CQN1 | Heat shock protein 75 kDa, mitochondrial | 242.62 | 1.68 |
| P02088 | Hemoglobin subunit beta-1 | 3543.4 | 1.67 |
| Q64525 | Histone H2B type 2-B | 1013.8 | 1.65 |
| Q64524 | Histone H2B type 2-E | 852.51 | 1.65 |
| P99024 | Tubulin beta-5 chain | 35888 | 1.65 |
| P70696 | Histone H2B type 1-A | 711.41 | 1.63 |
| P10853 | Histone H2B type 1-F/J/L | 1013.8 | 1.63 |
| Q64475 | Histone H2B type 1-B | 1013.8 | 1.60 |
| Q6ZWY9 | Histone H2B type 1-C/E/G | 1013.8 | 1.60 |
| Q8CGP1 | Histone H2B type 1-K | 1013.8 | 1.57 |
| P62806 | Histone H4 | 1669.8 | 1.54 |
| P68369 | Tubulin alpha-1A chain | 12360 | 1.52 |
| P02089 | Hemoglobin subunit beta-2 | 1972 | 1.51 |
| P17751 | Triosephosphate isomerase | 1422.4 | 1.49 |
| P68373 | Tubulin alpha-1C chain | 11309 | 1.49 |
| P16858 | Glyceraldehyde-3-phosphate dehydrogenase | 13052 | 1.48 |
| P08249 | Malate dehydrogenase, mitochondrial | 3024.4 | 1.48 |
| P11798 | Calcium/calmodulin-dependent protein kinase type II subunit alpha | 4430.8 | 1.46 |
| P50516 | V-type proton ATPase catalytic subunit A | 277.79 | 1.46 |
| P63268 | Actin, gamma-enteric smooth muscle | 24558 | 1.45 |
| P51881 | ADP/ATP translocase 2 | 102.17 | 1.45 |
| Q9JJZ2 | Tubulin alpha-8 chain | 4374.6 | 1.43 |
| P30275 | Creatine kinase U-type, mitochondrial | 521.45 | 1.40 |
| P29387 | Guanine nucleotide-binding protein subunit beta-4 | 984.78 | 1.40 |
| P07901 | Heat shock protein HSP 90-alpha | 283.32 | 1.38 |
| Q9CZU6 | Citrate synthase, mitochondrial | 136.89 | 1.36 |
| Q61011 | Guanine nucleotide-binding protein G(I)/G(S)/G(T) subunit beta-3 | 150.23 | 1.36 |
| P84091 | AP-2 complex subunit mu | 138.32 | 1.35 |
| P68134 | Actin, alpha skeletal muscle | 24717 | 1.34 |
| P62737 | Actin, aortic smooth muscle | 24552 | 1.34 |
| O55042 | Alpha-synuclein | 477.8 | 1.34 |
| Q9D6P8 | Calmodulin-like protein 3 | 89.23 | 1.34 |
| P07724 | Serum albumin | 102.93 | 1.34 |
| Q64332 | Synapsin-2 | 356.6 | 1.34 |
| P68033 | Actin, alpha cardiac muscle 1 | 24755 | 1.32 |
| P48962 | ADP/ATP translocase 1 | 173.37 | 1.31 |
| P14094 | Sodium/potassium-transporting ATPase subunit beta-1 | 604.37 | 1.31 |
| P28663 | Beta-soluble NSF attachment protein | 133.71 | 1.30 |
| P06745 | Glucose-6-phosphate isomerase | 613.13 | 1.30 |
| P62880 | Guanine nucleotide-binding protein G(I)/G(S)/G(T) subunit beta-2 | 1227.2 | 1.28 |
| P47857 | ATP-dependent 6-phosphofructokinase, muscle type | 66.63 | 1.26 |
| P17182 | Alpha-enolase | 4175.8 | 1.25 |
| Q64521 | Glycerol-3-phosphate dehydrogenase, mitochondrial | 81.8 | 1.25 |
| P70404 | Isocitrate dehydrogenase [NAD] subunit gamma 1, mitochondrial | 196.92 | 1.25 |
| P70296 | Phosphatidylethanolamine-binding protein 1 | 944.72 | 1.25 |
| Q6PIC6 | Sodium/potassium-transporting ATPase subunit alpha-3 | 1247.9 | 1.25 |
| Q91ZZ3 | Beta-synuclein | 76.97 | 1.23 |
| P43006 | Excitatory amino acid transporter 2 | 262.57 | 1.23 |
| P26443 | Glutamate dehydrogenase 1, mitochondrial | 717.95 | 1.23 |
| P01942 | Hemoglobin subunit alpha | 2208.1 | 1.23 |
| Q9Z0E0 | Neurochondrin | 107.25 | 1.23 |
| P63328 | Serine/threonine-protein phosphatase 2B catalytic subunit alpha isoform | 209.5 | 1.23 |
| Q6PIE5 | Sodium/potassium-transporting ATPase subunit alpha-2 | 1008.5 | 1.23 |
| Q9WV27 | Sodium/potassium-transporting ATPase subunit alpha-4 | 531.64 | 1.23 |
| Q03265 | ATP synthase subunit alpha, mitochondrial | 2101.7 | 1.22 |
| P39053 | Dynamin-1 | 4943.2 | 1.22 |
| P14152 | Malate dehydrogenase, cytoplasmic | 2371.9 | 1.22 |
| P62874 | Guanine nucleotide-binding protein G(I)/G(S)/G(T) subunit beta-1 | 996.97 | 1.21 |
| Q8VDN2 | Sodium/potassium-transporting ATPase subunit alpha-1 | 778.3 | 1.21 |
| Q9DB20 | ATP synthase subunit O, mitochondrial | 1284.3 | 1.20 |
| P0DP27 | Calmodulin-2 | 1448.3 | 1.20 |
| P0DP28 | Calmodulin-3 | 1448.3 | 1.20 |
| O08599 | Syntaxin-binding protein 1 | 1908.1 | 1.20 |
| Q8BZ98 | Dynamin-3 | 81.67 | 1.19 |
| P10126 | Elongation factor 1-alpha 1 | 355.16 | 1.19 |
| P05063 | Fructose-bisphosphate aldolase C | 782.97 | 1.19 |
| P31001 | Desmin | 114.92 | 1.17 |
| Q64478 | Histone H2B type 1-H | 1013.8 | 1.17 |
| P10854 | Histone H2B type 1-M | 1013.8 | 1.17 |
| Q8CGP2 | Histone H2B type 1-P | 1013.8 | 1.17 |
| P05213 | Tubulin alpha-1B chain | 12033 | 1.17 |
| P05201 | Aspartate aminotransferase, cytoplasmic | 401.64 | 1.16 |
| P21550 | Beta-enolase | 1943.9 | 1.16 |
| Q9D2U9 | Histone H2B type 3-A | 852.51 | 1.16 |
| P50396 | Rab GDP dissociation inhibitor alpha | 386.95 | 1.16 |
| Q9CZ13 | Cytochrome b-c1 complex subunit 1, mitochondrial | 232.11 | 1.15 |
| C0HKE7 | Histone H2A type 1-N | 1343.4 | 1.15 |
| Q8CGP0 | Histone H2B type 3-B | 852.51 | 1.15 |
| P05214 | Tubulin alpha-3 chain | 7666.1 | 1.15 |
| P68368 | Tubulin alpha-4A chain | 14150 | 1.15 |
| Q9CWF2 | Tubulin beta-2B chain | 37032 | 1.15 |
| P46460 | Vesicle-fusing ATPase | 225.46 | 1.15 |
| P39054 | Dynamin-2 | 80.91 | 1.14 |
| P08553 | Neurofilament medium polypeptide | 365.1 | 1.14 |
| P52480 | Pyruvate kinase PKM | 5072.3 | 1.14 |
| O88935 | Synapsin-1 | 752.24 | 1.14 |
| P68372 | Tubulin beta-4B chain | 28844 | 1.14 |
| Q60932 | Voltage-dependent anion-selective channel protein 1 | 1508.7 | 1.14 |
| Q68FD5 | Clathrin heavy chain 1 | 227.48 | 1.13 |
| P08551 | Neurofilament light polypeptide | 57.38 | 1.13 |
| P46096 | Synaptotagmin-1 | 213.16 | 1.13 |
| Q9ERD7 | Tubulin beta-3 chain | 25756 | 1.13 |
| Q6PHZ2 | Calcium/calmodulin-dependent protein kinase type II subunit delta | 1299.8 | 1.12 |
| P62814 | V-type proton ATPase subunit B, brain isoform | 95.93 | 1.12 |
| P17183 | Gamma-enolase | 3347.4 | 1.11 |
| P62631 | Elongation factor 1-alpha 2 | 167.42 | 1.09 |
| P99029 | Peroxiredoxin-5, mitochondrial | 1039.1 | 1.09 |
| P05202 | Aspartate aminotransferase, mitochondrial | 883.71 | 1.08 |
| P05064 | Fructose-bisphosphate aldolase A | 3093.5 | 1.08 |
| P16546 | Spectrin alpha chain, non-erythrocytic 1 | 133.67 | 1.08 |
| P28652 | Calcium/calmodulin-dependent protein kinase type II subunit beta | 1850.3 | 1.07 |
| P63101 | 14-3-3 protein zeta/delta | 4286.1 | 1.05 |
| Q62420 | Endophilin-A1 | 473.69 | -0.52 |
| Q8CGK7 | Guanine nucleotide-binding protein G(olf) subunit alpha | 1628.3 | -0.55 |
| Q62419 | Endophilin-A2 | 247.38 | -0.57 |
| P20801 | Troponin C, skeletal muscle | 394.5 | -0.57 |
| B2RSH2 | Guanine nucleotide-binding protein G(i) subunit alpha-1 | 1623.5 | -0.58 |
| Q9DC51 | Guanine nucleotide-binding protein G(i) subunit alpha | 1623.5 | -0.58 |
| P08752 | Guanine nucleotide-binding protein G(i) subunit alpha-2 | 1623.5 | -0.58 |
| P17156 | Heat shock-related 70 kDa protein 2 | 933.05 | -0.66 |
| Q91XV3 | Brain acid soluble protein 1 | 436.33 | -0.72 |
| P63044 | Vesicle-associated membrane protein 2 | 565.75 | -0.73 |
| P19783 | Cytochrome c oxidase subunit 4 isoform 1, mitochondrial | 321.26 | -0.78 |
| P16330 | 2',3'-cyclic-nucleotide 3'-phosphodiesterase | 887.7 | -0.83 |
| P46097 | Synaptotagmin-2 | 62.99 | -0.84 |
| P60202 | Myelin proteolipid protein | 5128.6 | -0.86 |
| P01831 | Thy-1 membrane glycoprotein | 202.4 | -0.86 |
| P16627 | Heat shock 70 kDa protein 1-like | 864.62 | -0.88 |
| Q62277 | Synaptophysin | 517.81 | -0.88 |
| P63017 | Heat shock cognate 71 kDa protein | 2484.2 | -0.90 |
| P04370 | Myelin basic protein | 9022.3 | -0.90 |
| O08553 | Dihydropyrimidinase-related protein 2 | 6913.4 | -0.91 |
| P18872 | Guanine nucleotide-binding protein G(o) subunit alpha | 2987 | -0.91 |
| P97427 | Dihydropyrimidinase-related protein 1 | 1567.8 | -0.94 |
| P63260 | Actin, cytoplasmic 2 | 32990 | -0.97 |
| Q3TXS7 | 26S proteasome non-ATPase regulatory subunit 1 | 114.46 | Control |
| Q9QXN3 | Activating signal cointegrator 1 | 64.68 | Control |
| E9Q394 | A-kinase anchor protein 13 | 49.13 | Control |
| Q9JII6 | Aldo-keto reductase family 1 member A1 | 122.11 | Control |
| Q91V80 | Apolipoprotein F | 151.7 | Control |
| O88879 | Apoptotic protease-activating factor 1 | 85 | Control |
| P39654 | Arachidonate 15-lipoxygenase | 45.59 | Control |
| P98203 | Armadillo repeat protein deleted in velo-cardio-facial syndrome homolog | 51.81 | Control |
| Q9DBR3 | Armadillo repeat-containing protein 8 | 35.27 | Control |
| Q91YH5 | Atlastin-3 | 105.62 | Control |
| Q9CQQ7 | ATP synthase F(0) complex subunit B1, mitochondrial | 126.25 | Control |
| P03930 | ATP synthase protein 8 | 681.06 | Control |
| Q9D3D9 | ATP synthase subunit delta, mitochondrial | 366.15 | Control |
| Q91VR2 | ATP synthase subunit gamma, mitochondrial | 148.77 | Control |
| E9PX95 | ATP-binding cassette sub-family A member 17 | 41.04 | Control |
| B5X0E4 | ATP-binding cassette sub-family B member 5 | 87.08 | Control |
| Q5PR69 | Cancer-related regulator of actin dynamics | 43.25 | Control |
| Q9CTG6 | Cation-transporting ATPase 13A2 | 70.86 | Control |
| Q5PR68 | Centrosomal protein of 112 kDa | 47.55 | Control |
| Q3UPP8 | Centrosomal protein of 63 kDa | 35.86 | Control |
| Q9Z0X4 | cGMP-inhibited 3',5'-cyclic phosphodiesterase A | 25.5 | Control |
| Q6ZPV2 | Chromatin-remodeling ATPase INO80 | 39.29 | Control |
| Q61548 | Clathrin coat assembly protein AP180 | 76.8 | Control |
| Q8CDV0 | Coiled-coil domain-containing protein 178 | 42.91 | Control |
| Q2UY11 | Collagen alpha-1(XXVIII) chain | 102.74 | Control |
| Q6DFV1 | Condensin-2 complex subunit G2 | 221.95 | Control |
| Q8BPN8 | DmX-like protein 2 | 27.86 | Control |
| Q8BHK9 | DNA excision repair protein ERCC-6-like | 16.41 | Control |
| P33611 | DNA polymerase alpha subunit B | 77.5 | Control |
| P70388 | DNA repair protein RAD50 | 47.24 | Control |
| Q8C7R7 | DNA-binding protein RFX6 | 56.61 | Control |
| Q3V0Q1 | Dynein heavy chain 12, axonemal | 42.8 | Control |
| Q91XQ0 | Dynein heavy chain 8, axonemal | 23.7 | Control |
| Q6ZQM0 | E3 ubiquitin-protein ligase rififylin | 69.42 | Control |
| Q5SQM0 | Echinoderm microtubule-associated protein-like 6 | 49.6 | Control |
| P08113 | Endoplasmin | 28.95 | Control |
| Q8R418 | Endoribonuclease Dicer | 45.65 | Control |
| Q3TDN2 | FAS-associated factor 2 | 115.28 | Control |
| Q922J9 | Fatty acyl-CoA reductase 1 | 64.67 | Control |
| Q80ZA4 | Fibrocystin-L | 23.87 | Control |
| Q8CI03 | FLYWCH-type zinc finger-containing protein 1 | 142.23 | Control |
| P49772 | Fms-related tyrosine kinase 3 ligand | 100.11 | Control |
| Q8K0C9 | GDP-mannose 4,6 dehydratase | 51.77 | Control |
| P97324 | Glucose-6-phosphate 1-dehydrogenase 2 | 38.33 | Control |
| Q9D4P7 | Glutathione S-transferase theta-4 | 127.3 | Control |
| Q3UFB7 | High affinity nerve growth factor receptor | 46.22 | Control |
| Q8C2B3 | Histone deacetylase 7 | 46.29 | Control |
| Q63ZW7 | InaD-like protein | 108.24 | Control |
| P56477 | Interferon regulatory factor 5 | 136.19 | Control |
| A2CG49 | Kalirin | 47.52 | Control |
| Q6IME9 | Keratin, type II cytoskeletal 72 | 52.82 | Control |
| P28738 | Kinesin heavy chain isoform 5C | 49.49 | Control |
| Q3URE9 | Leucine-rich repeat and immunoglobulin-like domain-containing nogo receptor-interacting protein 2 | 106.78 | Control |
| P51885 | Lumican | 87.64 | Control |
| Q80U28 | MAP kinase-activating death domain protein | 82.2 | Control |
| P53349 | Mitogen-activated protein kinase kinase kinase 1 | 42.29 | Control |
| Q9D6J5 | NADH dehydrogenase [ubiquinone] 1 beta subcomplex subunit 8, mitochondrial | 74.34 | Control |
| Q9DCT2 | NADH dehydrogenase [ubiquinone] iron-sulfur protein 3, mitochondrial | 105.89 | Control |
| Q8CH77 | Neuron navigator 1 | 37.69 | Control |
| Q6DFV7 | Nuclear receptor coactivator 7 | 55.17 | Control |
| Q8K4K6 | Pantothenate kinase 1 | 56.14 | Control |
| Q8R1G6 | PDZ and LIM domain protein 2 | 54.81 | Control |
| O70167 | Phosphatidylinositol 4-phosphate 3-kinase C2 domain-containing subunit gamma | 116.13 | Control |
| Q8CGT6 | Piwi-like protein 4 | 77.12 | Control |
| Q7TQ62 | Podocan | 87.39 | Control |
| Q91Z31 | Polypyrimidine tract-binding protein 2 | 53.52 | Control |
| Q99MQ1 | Protein bicaudal C homolog 1 | 60.29 | Control |
| P27773 | Protein disulfide-isomerase A3 | 57.17 | Control |
| Q9DAI6 | Protein FAM135B | 46.17 | Control |
| E9Q8I9 | Protein furry homolog | 49.48 | Control |
| Q61644 | Protein kinase C and casein kinase substrate in neurons protein 1 | 140.75 | Control |
| Q8R1F1 | Protein Niban 2 | 87.89 | Control |
| Q8K3V4 | Protein-arginine deiminase type-6 | 54.78 | Control |
| Q91Y02 | Protocadherin beta-18 | 65 | Control |
| Q8VCD6 | Receptor expression-enhancing protein 2 | 103.82 | Control |
| Q62132 | Receptor-type tyrosine-protein phosphatase R | 43.05 | Control |
| B9EKR1 | Receptor-type tyrosine-protein phosphatase zeta | 21.41 | Control |
| Q8K0T0 | Reticulon-1 | 68.24 | Control |
| Q80U35 | Rho guanine nucleotide exchange factor 17 | 48.63 | Control |
| Q9D3G9 | Rho-related GTP-binding protein RhoH | 86.75 | Control |
| Q3UZ01 | RNA-binding region-containing protein 3 | 100.41 | Control |
| P60330 | Separin | 62.04 | Control |
| Q91ZR4 | Serine/threonine-protein kinase Nek8 | 39.5 | Control |
| Q9JK88 | Serpin I2 | 57.22 | Control |
| Q60665 | Ski-like protein | 41.91 | Control |
| Q8K596 | Sodium/calcium exchanger 2 | 64.72 | Control |
| Q8K078 | Solute carrier organic anion transporter family member 4A1 | 58.66 | Control |
| Q9D3S3 | Sorting nexin-29 | 26.68 | Control |
| A0AUV4 | Sperm motility kinase Y | 79.3 | Control |
| Q5U4C3 | Splicing factor, arginine/serine-rich 19 | 29.48 | Control |
| F6XZJ7 | Sterile alpha motif domain-containing protein 15 | 44.9 | Control |
| P83093 | Stromal interaction molecule 2 | 48.68 | Control |
| Q9WUM5 | Succinate--CoA ligase [ADP/GDP-forming] subunit alpha, mitochondrial | 340.57 | Control |
| O55100 | Synaptogyrin-1 | 262.69 | Control |
| P80316 | T-complex protein 1 subunit epsilon | 200.72 | Control |
| P42932 | T-complex protein 1 subunit theta | 149.24 | Control |
| Q80W22 | Threonine synthase-like 2 | 96.5 | Control |
| Q9Z1T2 | Thrombospondin-4 | 52.59 | Control |
| Q61286 | Transcription factor 12 | 98.9 | Control |
| Q6QR59 | TRPM8 channel-associated factor 3 | 290.23 | Control |
| Q9D906 | Ubiquitin-like modifier-activating enzyme ATG7 | 56.75 | Control |
| P40336 | Vacuolar protein sorting-associated protein 26A | 139.42 | Control |
| Q62059 | Versican core protein | 63.41 | Control |
| P29788 | Vitronectin | 55.89 | Control |
| P46471 | 26S proteasome regulatory subunit 7 | 47.83 | 50 mg/L |
| P61922 | 4-aminobutyrate aminotransferase, mitochondrial | 391.09 | 50 mg/L |
| P10852 | 4F2 cell-surface antigen heavy chain | 152.62 | 50 mg/L |
| P61161 | Actin-related protein 2 | 100.9 | 50 mg/L |
| Q9WV32 | Actin-related protein 2/3 complex subunit 1B | 83.28 | 50 mg/L |
| Q9CVB6 | Actin-related protein 2/3 complex subunit 2 | 216.14 | 50 mg/L |
| Q9JM76 | Actin-related protein 2/3 complex subunit 3 | 574.56 | 50 mg/L |
| Q61271 | Activin receptor type-1B | 44.62 | 50 mg/L |
| P31786 | Acyl-CoA-binding protein | 1758.3 | 50 mg/L |
| Q9R0Y5 | Adenylate kinase isoenzyme 1 | 383.65 | 50 mg/L |
| P84078 | ADP-ribosylation factor 1 | 78.16 | 50 mg/L |
| Q8BSL7 | ADP-ribosylation factor 2 | 54.21 | 50 mg/L |
| P61205 | ADP-ribosylation factor 3 | 78.16 | 50 mg/L |
| P61750 | ADP-ribosylation factor 4 | 416.87 | 50 mg/L |
| P84084 | ADP-ribosylation factor 5 | 416.87 | 50 mg/L |
| Q7TPR4 | Alpha-actinin-1 | 59.63 | 50 mg/L |
| Q9QYC0 | Alpha-adducin | 266.87 | 50 mg/L |
| Q920R0 | Alsin | 139.19 | 50 mg/L |
| Q7TQF7 | Amphiphysin | 110.92 | 50 mg/L |
| Q7TQI7 | Ankyrin repeat and BTB/POZ domain-containing protein 2 | 93.72 | 50 mg/L |
| Q8BZ05 | Arf-GAP with Rho-GAP domain, ANK repeat and PH domain-containing protein 2 | 24.9 | 50 mg/L |
| Q497K5 | Arrestin domain-containing protein 5 | 80.25 | 50 mg/L |
| Q8R3P0 | Aspartoacylase | 61.32 | 50 mg/L |
| Q9DCX2 | ATP synthase subunit d, mitochondrial | 768.46 | 50 mg/L |
| E9Q4N7 | AT-rich interactive domain-containing protein 1B | 21.9 | 50 mg/L |
| Q61361 | Brevican core protein | 66.41 | 50 mg/L |
| P12367 | cAMP-dependent protein kinase type II-alpha regulatory subunit | 204.71 | 50 mg/L |
| P31324 | cAMP-dependent protein kinase type II-beta regulatory subunit | 204.71 | 50 mg/L |
| B9EHT4 | CAP-Gly domain-containing linker protein 3 | 52.39 | 50 mg/L |
| Q65CL1 | Catenin alpha-3 | 41.45 | 50 mg/L |
| Q5M8N0 | CB1 cannabinoid receptor-interacting protein 1 | 269.5 | 50 mg/L |
| Q91WS0 | CDGSH iron-sulfur domain-containing protein 1 | 189.08 | 50 mg/L |
| Q640L3 | Cell cycle progression protein 1 | 60.82 | 50 mg/L |
| Q7TSH4 | Centriolar coiled-coil protein of 110 kDa | 66.84 | 50 mg/L |
| Q62036 | Centrosomal protein of 131 kDa | 44.53 | 50 mg/L |
| E9Q309 | Centrosome-associated protein 350 | 26.11 | 50 mg/L |
| E9PZM4 | Chromodomain-helicase-DNA-binding protein 2 | 375.14 | 50 mg/L |
| Q80YR7 | Claspin | 89.03 | 50 mg/L |
| P63040 | Complexin-1 | 49.54 | 50 mg/L |
| P84086 | Complexin-2 | 253.93 | 50 mg/L |
| Q9DB77 | Cytochrome b-c1 complex subunit 2, mitochondrial | 149.5 | 50 mg/L |
| P56391 | Cytochrome c oxidase subunit 6B1 | 85.72 | 50 mg/L |
| O55071 | Cytochrome P450 2B19 | 63.07 | 50 mg/L |
| Q9CX98 | Cytochrome P450 2U1 | 59.31 | 50 mg/L |
| O35728 | Cytochrome P450 4A14 | 65.59 | 50 mg/L |
| Q99KU1 | Dehydrodolichyl diphosphate synthase complex subunit Dhdds | 105.52 | 50 mg/L |
| Q9EQF6 | Dihydropyrimidinase-related protein 5 | 82.47 | 50 mg/L |
| Q80U19 | Disheveled-associated activator of morphogenesis 2 | 38.52 | 50 mg/L |
| Q8K4R9 | Disks large-associated protein 5 | 54.05 | 50 mg/L |
| Q61881 | DNA replication licensing factor MCM7 | 53.8 | 50 mg/L |
| Q8CJ67 | Double-stranded RNA-binding protein Staufen homolog 2 | 90.3 | 50 mg/L |
| Q8R0K2 | E3 ubiquitin-protein ligase TRIM31 | 80.78 | 50 mg/L |
| Q8BFR5 | Elongation factor Tu, mitochondrial | 62.53 | 50 mg/L |
| P56564 | Excitatory amino acid transporter 1 | 143.9 | 50 mg/L |
| Q9ERK4 | Exportin-2 | 79.65 | 50 mg/L |
| Q9WVH3 | Forkhead box protein O4 | 75.41 | 50 mg/L |
| Q8C0M0 | GATOR complex protein WDR59 | 31.1 | 50 mg/L |
| Q9ESZ8 | General transcription factor II-I | 115.83 | 50 mg/L |
| P19157 | Glutathione S-transferase P 1 | 238.47 | 50 mg/L |
| P55937 | Golgin subfamily A member 3 | 42.33 | 50 mg/L |
| Q91VW5 | Golgin subfamily A member 4 | 65.52 | 50 mg/L |
| Q8K349 | GTPase IMAP family member 6 | 69.55 | 50 mg/L |
| Q9R0C8 | Guanine nucleotide exchange factor VAV3 | 33.25 | 50 mg/L |
| Q61316 | Heat shock 70 kDa protein 4 | 88.69 | 50 mg/L |
| Q61699 | Heat shock protein 105 kDa | 156.6 | 50 mg/L |
| P22361 | Hepatocyte nuclear factor 1-alpha | 72.1 | 50 mg/L |
| P61979 | Heterogeneous nuclear ribonucleoprotein K | 413.21 | 50 mg/L |
| Q8R081 | Heterogeneous nuclear ribonucleoprotein L | 125.22 | 50 mg/L |
| Q8VEK3 | Heterogeneous nuclear ribonucleoprotein U | 63.33 | 50 mg/L |
| O88569 | Heterogeneous nuclear ribonucleoproteins A2/B1 | 168.19 | 50 mg/L |
| Q91W97 | Hexokinase HKDC1 | 357.2 | 50 mg/L |
| O08528 | Hexokinase-2 | 344.36 | 50 mg/L |
| Q8R366 | Immunoglobulin superfamily member 8 | 77.23 | 50 mg/L |
| Q61739 | Integrin alpha-6 | 56.16 | 50 mg/L |
| Q60677 | Integrin alpha-E | 55.64 | 50 mg/L |
| Q8BJD1 | Inter-alpha-trypsin inhibitor heavy chain H5 | 33.53 | 50 mg/L |
| Q9QY61 | Iroquois-class homeodomain protein IRX-4 | 116.02 | 50 mg/L |
| O54983 | Ketimine reductase mu-crystallin | 437.71 | 50 mg/L |
| P02468 | Laminin subunit gamma-1 | 85.81 | 50 mg/L |
| Q8C0R9 | Leucine-rich repeat and death domain-containing protein 1 | 50.31 | 50 mg/L |
| A2AWL7 | MAX gene-associated protein | 22.4 | 50 mg/L |
| Q8BJS8 | Mdm2-binding protein | 33.47 | 50 mg/L |
| B1AYB6 | Methyl-CpG-binding domain protein 5 | 130.49 | 50 mg/L |
| Q9QYR6 | Microtubule-associated protein 1A | 26 | 50 mg/L |
| P54279 | Mismatch repair endonuclease PMS2 | 81.88 | 50 mg/L |
| Q9D6M3 | Mitochondrial glutamate carrier 1 | 935.32 | 50 mg/L |
| Q9DB41 | Mitochondrial glutamate carrier 2 | 261.54 | 50 mg/L |
| Q63844 | Mitogen-activated protein kinase 3 | 44.51 | 50 mg/L |
| Q61885 | Myelin-oligodendrocyte glycoprotein | 394.33 | 50 mg/L |
| Q99MD8 | Myoneurin | 59.94 | 50 mg/L |
| Q9D6J6 | NADH dehydrogenase [ubiquinone] flavoprotein 2, mitochondrial | 275.26 | 50 mg/L |
| Q91VD9 | NADH-ubiquinone oxidoreductase 75 kDa subunit, mitochondrial | 68.23 | 50 mg/L |
| Q91V57 | N-chimaerin | 127.8 | 50 mg/L |
| P06837 | Neuromodulin | 330.94 | 50 mg/L |
| P35802 | Neuronal membrane glycoprotein M6-a | 62.54 | 50 mg/L |
| P97460 | Neuronal PAS domain-containing protein 2 | 157.65 | 50 mg/L |
| P97300 | Neuroplastin | 257.58 | 50 mg/L |
| Q61043 | Ninein | 34.85 | 50 mg/L |
| Q8R5A0 | N-lysine methyltransferase SMYD2 | 40.64 | 50 mg/L |
| Q02780 | Nuclear factor 1 A-type | 129.75 | 50 mg/L |
| P56716 | Oxygen-regulated protein 1 | 43.45 | 50 mg/L |
| P35700 | Peroxiredoxin-1 | 86.99 | 50 mg/L |
| O08807 | Peroxiredoxin-4 | 57.42 | 50 mg/L |
| Q9D4H9 | PHD finger protein 14 | 68.53 | 50 mg/L |
| P53811 | Phosphatidylinositol transfer protein beta isoform | 83.52 | 50 mg/L |
| Q9R0K7 | Plasma membrane calcium-transporting ATPase 2 | 77.91 | 50 mg/L |
| P26618 | Platelet-derived growth factor receptor alpha | 48.55 | 50 mg/L |
| P0CG49 | Polyubiquitin-B | 8981.6 | 50 mg/L |
| P0CG50 | Polyubiquitin-C | 8986.8 | 50 mg/L |
| P67778 | Prohibitin | 112.15 | 50 mg/L |
| Q6TDU8 | Protein CASC1 | 29.28 | 50 mg/L |
| Q2EMV9 | Protein mono-ADP-ribosyltransferase PARP14 | 33.61 | 50 mg/L |
| O55125 | Protein NipSnap homolog 1 | 128.44 | 50 mg/L |
| Q3UYC0 | Protein phosphatase 1H | 136.49 | 50 mg/L |
| G3UYX5 | Regulator of G-protein signaling 22 | 61.47 | 50 mg/L |
| P70336 | Rho-associated protein kinase 2 | 37.51 | 50 mg/L |
| Q9D7H3 | RNA 3'-terminal phosphate cyclase | 151.46 | 50 mg/L |
| A2AGL3 | Ryanodine receptor 3 | 31.45 | 50 mg/L |
| Q8C650 | Septin-10 | 112.05 | 50 mg/L |
| Q8C1B7 | Septin-11 | 118.18 | 50 mg/L |
| Q9DA97 | Septin-14 | 112.05 | 50 mg/L |
| Q9R1T4 | Septin-6 | 290.38 | 50 mg/L |
| Q91V61 | Sideroflexin-3 | 115.13 | 50 mg/L |
| P52019 | Squalene monooxygenase | 113.26 | 50 mg/L |
| Q99JB2 | Stomatin-like protein 2, mitochondrial | 113.32 | 50 mg/L |
| Q9ERG2 | Striatin-3 | 44.66 | 50 mg/L |
| Q5SVR0 | TBC1 domain family member 9B | 32.16 | 50 mg/L |
| Q7TN22 | Thioredoxin domain-containing protein 16 | 86.01 | 50 mg/L |
| P70399 | TP53-binding protein 1 | 100.25 | 50 mg/L |
| P24529 | Tyrosine 3-monooxygenase | 133.86 | 50 mg/L |
| P62983 | Ubiquitin-40S ribosomal protein S27a | 8981.6 | 50 mg/L |
| P62984 | Ubiquitin-60S ribosomal protein L40 | 8981.6 | 50 mg/L |
| A2RSJ4 | UHRF1-binding protein 1-like | 334.57 | 50 mg/L |
| Q3U2A8 | Valine--tRNA ligase, mitochondrial | 33.7 | 50 mg/L |
| P62761 | Visinin-like protein 1 | 102.26 | 50 mg/L |
| Q60931 | Voltage-dependent anion-selective channel protein 3 | 104.66 | 50 mg/L |
| P51863 | V-type proton ATPase subunit d 1 | 140.32 | 50 mg/L |
| P50518 | V-type proton ATPase subunit E 1 | 373.39 | 50 mg/L |
| Q8BVE3 | V-type proton ATPase subunit H | 355.93 | 50 mg/L |
| Q8K1X1 | WD repeat-containing protein 11 | 52.43 | 50 mg/L |

ªUniprot accession ID retrieved from uniport.org database; Negative values of fold change mean down-regulated proteins; 50 mg/L or Control in fold change column means that protein was exclusively found in the respective experimental.

**Supplementary table 4.** Identified proteins with expression significantly altered in the hippocampus of mice 50 mg/L group vs. 10 mg/L group.

| Accession IDª | Protein Description | PLGS  Score | Fold change |
| --- | --- | --- | --- |
| A2AQ07 | Tubulin beta-1 chain | 168.6 | 5.99 |
| P02104 | Hemoglobin subunit epsilon-Y2 | 2642.5 | 5.21 |
| Q61595 | Kinectin | 92.07 | 4.9 |
| P02088 | Hemoglobin subunit beta-1 | 2936.3 | 4.53 |
| P01942 | Hemoglobin subunit alpha | 1210.9 | 3.6 |
| P68372 | Tubulin beta-4B chain | 33674 | 2.75 |
| Q9D6F9 | Tubulin beta-4A chain | 16336 | 2.69 |
| Q8BZQ7 | Anaphase-promoting complex subunit 2 | 87.56 | 2.66 |
| P99024 | Tubulin beta-5 chain | 39072 | 2.64 |
| P61164 | Alpha-centractin | 397.13 | 2.59 |
| Q6PHZ2 | Calcium/calmodulin-dependent protein kinase type II subunit delta | 574.52 | 2.59 |
| P16858 | Glyceraldehyde-3-phosphate dehydrogenase | 8044 | 2.59 |
| Q64524 | Histone H2B type 2-E | 2291.7 | 2.53 |
| Q7TMM9 | Tubulin beta-2A chain | 39321 | 2.53 |
| Q8R5C5 | Beta-centractin | 408.8 | 2.48 |
| Q9CWF2 | Tubulin beta-2B chain | 39241 | 2.44 |
| Q9Z1W8 | Potassium-transporting ATPase alpha chain 2 | 299.69 | 2.41 |
| Q9WV27 | Sodium/potassium-transporting ATPase subunit alpha-4 | 309.34 | 2.41 |
| Q923T9 | Calcium/calmodulin-dependent protein kinase type II subunit gamma | 574.52 | 2.36 |
| P68134 | Actin, alpha skeletal muscle | 29792 | 2.27 |
| P62737 | Actin, aortic smooth muscle | 29766 | 2.27 |
| P68033 | Actin, alpha cardiac muscle 1 | 29792 | 2.25 |
| Q9D2U9 | Histone H2B type 3-A | 2291.7 | 2.25 |
| P63268 | Actin, gamma-enteric smooth muscle | 29766 | 2.23 |
| P10126 | Elongation factor 1-alpha 1 | 326.72 | 2.03 |
| P60710 | Actin, cytoplasmic 1 | 40198 | 1.95 |
| Q04447 | Creatine kinase B-type | 1452.3 | 1.95 |
| Q922F4 | Tubulin beta-6 chain | 21599 | 1.92 |
| P11798 | Calcium/calmodulin-dependent protein kinase type II subunit alpha | 2519.4 | 1.82 |
| P53657 | Pyruvate kinase PKLR | 62.8 | 1.82 |
| P16330 | 2',3'-cyclic-nucleotide 3'-phosphodiesterase | 417.65 | 1.79 |
| Q8BFZ3 | Beta-actin-like protein 2 | 7523.9 | 1.79 |
| P63260 | Actin, cytoplasmic 2 | 40198 | 1.77 |
| Q61696 | Heat shock 70 kDa protein 1A | 643.25 | 1.7 |
| P17879 | Heat shock 70 kDa protein 1B | 643.25 | 1.67 |
| P70696 | Histone H2B type 1-A | 333.92 | 1.67 |
| P04370 | Myelin basic protein | 2857 | 1.63 |
| P60202 | Myelin proteolipid protein | 4035.3 | 1.63 |
| P08553 | Neurofilament medium polypeptide | 146.5 | 1.63 |
| P18872 | Guanine nucleotide-binding protein G(o) subunit alpha | 2969.4 | 1.62 |
| Q03265 | ATP synthase subunit alpha, mitochondrial | 1417.1 | 1.6 |
| P0DP28 | Calmodulin-3 | 830.62 | 1.6 |
| P15105 | Glutamine synthetase | 424.79 | 1.6 |
| P0DP27 | Calmodulin-2 | 830.62 | 1.58 |
| Q62277 | Synaptophysin | 940.78 | 1.58 |
| P0DP26 | Calmodulin-1 | 830.62 | 1.57 |
| P16627 | Heat shock 70 kDa protein 1-like | 656.84 | 1.54 |
| Q9D6P8 | Calmodulin-like protein 3 | 1621.7 | 1.49 |
| Q9ERD7 | Tubulin beta-3 chain | 32419 | 1.49 |
| B2RSH2 | Guanine nucleotide-binding protein G(i) subunit alpha-1 | 2040.1 | 1.48 |
| P63017 | Heat shock cognate 71 kDa protein | 1105.3 | 1.48 |
| P17156 | Heat shock-related 70 kDa protein 2 | 754.3 | 1.48 |
| O88935 | Synapsin-1 | 247.5 | 1.48 |
| P18760 | Cofilin-1 | 1100.4 | 1.45 |
| O08553 | Dihydropyrimidinase-related protein 2 | 4617.4 | 1.43 |
| P08551 | Neurofilament light polypeptide | 277.72 | 1.43 |
| P31001 | Desmin | 57.06 | 1.42 |
| Q9DC51 | Guanine nucleotide-binding protein G(i) subunit alpha | 2040.1 | 1.42 |
| P62983 | Ubiquitin-40S ribosomal protein S27a | 1318.2 | 1.4 |
| P20152 | Vimentin | 103.93 | 1.4 |
| P46660 | Alpha-internexin | 299.92 | 1.39 |
| P08752 | Guanine nucleotide-binding protein G(i) subunit alpha-2 | 2040.1 | 1.39 |
| P0CG49 | Polyubiquitin-B | 1318.2 | 1.39 |
| P0CG50 | Polyubiquitin-C | 1318.2 | 1.39 |
| P62984 | Ubiquitin-60S ribosomal protein L40 | 1318.2 | 1.39 |
| P17742 | Peptidyl-prolyl cis-trans isomerase A | 1703.2 | 1.38 |
| P05063 | Fructose-bisphosphate aldolase C | 810.1 | 1.36 |
| P63094 | Guanine nucleotide-binding protein G(s) subunit alpha isoforms short | 2040.1 | 1.34 |
| P50149 | Guanine nucleotide-binding protein G(t) subunit alpha-2 | 2040.1 | 1.32 |
| P27600 | Guanine nucleotide-binding protein subunit alpha-12 | 2206 | 1.32 |
| Q8CGK7 | Guanine nucleotide-binding protein G(olf) subunit alpha | 2040.1 | 1.31 |
| Q6R0H7 | Guanine nucleotide-binding protein G(s) subunit alpha isoforms XLas | 2040.1 | 1.31 |
| P20612 | Guanine nucleotide-binding protein G(t) subunit alpha-1 | 2040.1 | 1.31 |
| Q3V3I2 | Guanine nucleotide-binding protein G(t) subunit alpha-3 | 2040.1 | 1.31 |
| P27601 | Guanine nucleotide-binding protein subunit alpha-13 | 2206 | 1.31 |
| P05064 | Fructose-bisphosphate aldolase A | 2667 | 1.26 |
| P60879 | Synaptosomal-associated protein 25 | 253.29 | 1.23 |
| P07724 | Serum albumin | 249.47 | 1.22 |
| P05213 | Tubulin alpha-1B chain | 14800 | 1.16 |
| P68369 | Tubulin alpha-1A chain | 14742 | 1.15 |
| P05214 | Tubulin alpha-3 chain | 8020.7 | 1.15 |
| P68373 | Tubulin alpha-1C chain | 13174 | 1.11 |
| P68368 | Tubulin alpha-4A chain | 12233 | 1.11 |
| P05202 | Aspartate aminotransferase, mitochondrial | 211.16 | 1.08 |
| Q68FD5 | Clathrin heavy chain 1 | 192.64 | 1.08 |
| P17183 | Gamma-enolase | 2014.2 | 1.07 |
| A2AL36 | Centriolin | 70.22 | -0.28 |
| Q64467 | Glyceraldehyde-3-phosphate dehydrogenase, testis-specific | 534.1 | -0.41 |
| Q9CQN1 | Heat shock protein 75 kDa, mitochondrial | 1035.3 | -0.5 |
| P20801 | Troponin C, skeletal muscle | 557.51 | -0.52 |
| P50518 | V-type proton ATPase subunit E 1 | 302.13 | -0.53 |
| Q91VD9 | NADH-ubiquinone oxidoreductase 75 kDa subunit, mitochondrial | 303.78 | -0.56 |
| P06837 | Neuromodulin | 347.57 | -0.58 |
| Q9Z2Q6 | Septin-5 | 356.87 | -0.58 |
| O08749 | Dihydrolipoyl dehydrogenase, mitochondrial | 83.56 | -0.59 |
| Q61011 | Guanine nucleotide-binding protein G(I)/G(S)/G(T) subunit beta-3 | 1057.6 | -0.59 |
| Q8CGP6 | Histone H2A type 1-H | 566.36 | -0.59 |
| C0HKE1 | Histone H2A type 1-B | 566.36 | -0.61 |
| C0HKE4 | Histone H2A type 1-E | 566.36 | -0.61 |
| Q6GSS7 | Histone H2A type 2-A | 566.36 | -0.61 |
| Q64523 | Histone H2A type 2-C | 566.36 | -0.61 |
| Q8R1M2 | Histone H2A.J | 566.36 | -0.61 |
| P62806 | Histone H4 | 3283.9 | -0.61 |
| P29387 | Guanine nucleotide-binding protein subunit beta-4 | 3728.7 | -0.62 |
| C0HKE3 | Histone H2A type 1-D | 566.36 | -0.62 |
| Q8CGP5 | Histone H2A type 1-F | 566.36 | -0.62 |
| C0HKE5 | Histone H2A type 1-G | 566.36 | -0.62 |
| C0HKE7 | Histone H2A type 1-N | 566.36 | -0.62 |
| C0HKE8 | Histone H2A type 1-O | 566.36 | -0.62 |
| C0HKE9 | Histone H2A type 1-P | 566.36 | -0.62 |
| Q8BFU2 | Histone H2A type 3 | 566.36 | -0.62 |
| P63101 | 14-3-3 protein zeta/delta | 4355.7 | -0.63 |
| Q9CR68 | Cytochrome b-c1 complex subunit Rieske, mitochondrial | 862.55 | -0.63 |
| C0HKE2 | Histone H2A type 1-C | 566.36 | -0.63 |
| C0HKE6 | Histone H2A type 1-I | 566.36 | -0.63 |
| Q8CGP7 | Histone H2A type 1-K | 566.36 | -0.63 |
| Q8BMF4 | Dihydrolipoyllysine-residue acetyltransferase component of pyruvate dehydrogenase complex, mitochondrial | 157.68 | -0.64 |
| P08228 | Superoxide dismutase [Cu-Zn] | 561.16 | -0.65 |
| P62259 | 14-3-3 protein epsilon | 3377.9 | -0.66 |
| P68254 | 14-3-3 protein theta | 3427.4 | -0.66 |
| Q91XV3 | Brain acid soluble protein 1 | 334.05 | -0.66 |
| P20029 | Endoplasmic reticulum chaperone BiP | 1685.2 | -0.66 |
| P16125 | L-lactate dehydrogenase B chain | 1913.3 | -0.66 |
| Q9QYR6 | Microtubule-associated protein 1A | 42.54 | -0.66 |
| Q9CQV8 | 14-3-3 protein beta/alpha | 3457.3 | -0.67 |
| P61982 | 14-3-3 protein gamma | 4013.2 | -0.67 |
| P62880 | Guanine nucleotide-binding protein G(I)/G(S)/G(T) subunit beta-2 | 4153.3 | -0.67 |
| P28663 | Beta-soluble NSF attachment protein | 448.22 | -0.68 |
| P19783 | Cytochrome c oxidase subunit 4 isoform 1, mitochondrial | 1353.2 | -0.68 |
| Q62420 | Endophilin-A1 | 534.27 | -0.68 |
| P07901 | Heat shock protein HSP 90-alpha | 1228 | -0.68 |
| Q3UX10 | Tubulin alpha chain-like 3 | 235.27 | -0.68 |
| Q8BVE3 | V-type proton ATPase subunit H | 172.23 | -0.68 |
| Q64433 | 10 kDa heat shock protein, mitochondrial | 733.54 | -0.69 |
| O70456 | 14-3-3 protein sigma | 3382.1 | -0.69 |
| P51881 | ADP/ATP translocase 2 | 492.15 | -0.69 |
| Q3V132 | ADP/ATP translocase 4 | 274.71 | -0.69 |
| Q01768 | Nucleoside diphosphate kinase B | 750.12 | -0.69 |
| Q91ZZ3 | Beta-synuclein | 3392 | -0.7 |
| P62874 | Guanine nucleotide-binding protein G(I)/G(S)/G(T) subunit beta-1 | 4165.7 | -0.7 |
| P00342 | L-lactate dehydrogenase C chain | 1010.2 | -0.7 |
| P46097 | Synaptotagmin-2 | 124.21 | -0.7 |
| P68510 | 14-3-3 protein eta | 3427.4 | -0.71 |
| Q8BSL7 | ADP-ribosylation factor 2 | 1134.6 | -0.71 |
| Q9DB77 | Cytochrome b-c1 complex subunit 2, mitochondrial | 430.39 | -0.71 |
| P08249 | Malate dehydrogenase, mitochondrial | 1459.2 | -0.71 |
| Q9D051 | Pyruvate dehydrogenase E1 component subunit beta, mitochondrial | 145.38 | -0.71 |
| P14094 | Sodium/potassium-transporting ATPase subunit beta-1 | 3370.7 | -0.71 |
| Q64475 | Histone H2B type 1-B | 2822.6 | -0.72 |
| P10853 | Histone H2B type 1-F/J/L | 2822.6 | -0.72 |
| Q64525 | Histone H2B type 2-B | 2822.6 | -0.72 |
| Q9DD03 | Ras-related protein Rab-13 | 308.37 | -0.72 |
| Q9R0N5 | Synaptotagmin-5 | 93.86 | -0.72 |
| Q6ZWY9 | Histone H2B type 1-C/E/G | 2822.6 | -0.73 |
| Q64478 | Histone H2B type 1-H | 2822.6 | -0.73 |
| Q8CGP1 | Histone H2B type 1-K | 2822.6 | -0.73 |
| P10854 | Histone H2B type 1-M | 2822.6 | -0.73 |
| Q8CGP2 | Histone H2B type 1-P | 2822.6 | -0.73 |
| Q8CGP0 | Histone H2B type 3-B | 2291.7 | -0.73 |
| P35802 | Neuronal membrane glycoprotein M6-a | 67.63 | -0.73 |
| P70296 | Phosphatidylethanolamine-binding protein 1 | 2276.6 | -0.73 |
| Q64436 | Potassium-transporting ATPase alpha chain 1 | 20.73 | -0.73 |
| Q64332 | Synapsin-2 | 143.9 | -0.73 |
| P06151 | L-lactate dehydrogenase A chain | 1477.7 | -0.74 |
| P01831 | Thy-1 membrane glycoprotein | 388.2 | -0.74 |
| P84078 | ADP-ribosylation factor 1 | 1615.3 | -0.75 |
| P61205 | ADP-ribosylation factor 3 | 1615.3 | -0.75 |
| P48962 | ADP/ATP translocase 1 | 640.34 | -0.76 |
| P20357 | Microtubule-associated protein 2 | 398.89 | -0.76 |
| P46096 | Synaptotagmin-1 | 467.04 | -0.76 |
| P31786 | Acyl-CoA-binding protein | 1471.6 | -0.77 |
| P47857 | ATP-dependent 6-phosphofructokinase, muscle type | 386.14 | -0.78 |
| P02089 | Hemoglobin subunit beta-2 | 2642.5 | -0.78 |
| Q9DBJ1 | Phosphoglycerate mutase 1 | 1170.7 | -0.78 |
| O55042 | Alpha-synuclein | 1500.1 | -0.79 |
| P56480 | ATP synthase subunit beta, mitochondrial | 2086.2 | -0.79 |
| P00405 | Cytochrome c oxidase subunit 2 | 282.96 | -0.79 |
| P12787 | Cytochrome c oxidase subunit 5A, mitochondrial | 2092.4 | -0.79 |
| P26443 | Glutamate dehydrogenase 1, mitochondrial | 120.26 | -0.79 |
| S4R2M7 | Phosphoglycerate kinase | 90.57 | -0.79 |
| Q61598 | Rab GDP dissociation inhibitor beta | 879.55 | -0.79 |
| Q62261 | Spectrin beta chain, non-erythrocytic 1 | 218.26 | -0.79 |
| O08599 | Syntaxin-binding protein 1 | 1513 | -0.79 |
| P17710 | Hexokinase-1 | 96.46 | -0.8 |
| P17751 | Triosephosphate isomerase | 696.18 | -0.8 |
| Q60930 | Voltage-dependent anion-selective channel protein 2 | 741.88 | -0.8 |
| P10649 | Glutathione S-transferase Mu 1 | 1091.2 | -0.81 |
| Q02053 | Ubiquitin-like modifier-activating enzyme 1 | 102.99 | -0.81 |
| Q8BZ98 | Dynamin-3 | 288.11 | -0.82 |
| P15532 | Nucleoside diphosphate kinase A | 999.05 | -0.82 |
| P09041 | Phosphoglycerate kinase 2 | 101.21 | -0.82 |
| Q9D6R2 | Isocitrate dehydrogenase [NAD] subunit alpha, mitochondrial | 98.41 | -0.83 |
| Q8VEM8 | Phosphate carrier protein, mitochondrial | 425.26 | -0.83 |
| P55258 | Ras-related protein Rab-8A | 1045.2 | -0.83 |
| P39054 | Dynamin-2 | 296.96 | -0.84 |
| P62631 | Elongation factor 1-alpha 2 | 51.32 | -0.84 |
| P43006 | Excitatory amino acid transporter 2 | 1126 | -0.84 |
| P06745 | Glucose-6-phosphate isomerase | 593.01 | -0.84 |
| Q61171 | Peroxiredoxin-2 | 1065.8 | -0.84 |
| P63011 | Ras-related protein Rab-3A | 2770.6 | -0.84 |
| Q9CZT8 | Ras-related protein Rab-3B | 2222.7 | -0.84 |
| P62823 | Ras-related protein Rab-3C | 2222.7 | -0.84 |
| P35276 | Ras-related protein Rab-3D | 2349 | -0.84 |
| P61028 | Ras-related protein Rab-8B | 1045.2 | -0.84 |
| P48453 | Serine/threonine-protein phosphatase 2B catalytic subunit beta isoform | 206.19 | -0.84 |
| Q8VDN2 | Sodium/potassium-transporting ATPase subunit alpha-1 | 299.61 | -0.84 |
| Q6PIE5 | Sodium/potassium-transporting ATPase subunit alpha-2 | 391.86 | -0.84 |
| Q60932 | Voltage-dependent anion-selective channel protein 1 | 1285.5 | -0.84 |
| Q9DB20 | ATP synthase subunit O, mitochondrial | 3233.6 | -0.85 |
| P14152 | Malate dehydrogenase, cytoplasmic | 1010.9 | -0.85 |
| P50396 | Rab GDP dissociation inhibitor alpha | 1806.3 | -0.85 |
| P63328 | Serine/threonine-protein phosphatase 2B catalytic subunit alpha isoform | 464.27 | -0.85 |
| Q8K386 | Ras-related protein Rab-15 | 905.96 | -0.86 |
| P62821 | Ras-related protein Rab-1A | 928.3 | -0.86 |
| P28652 | Calcium/calmodulin-dependent protein kinase type II subunit beta | 608.48 | -0.87 |
| P09411 | Phosphoglycerate kinase 1 | 90.57 | -0.87 |
| Q6PIC6 | Sodium/potassium-transporting ATPase subunit alpha-3 | 461.07 | -0.87 |
| P50516 | V-type proton ATPase catalytic subunit A | 269.63 | -0.87 |
| P17182 | Alpha-enolase | 2930.6 | -0.88 |
| Q62188 | Dihydropyrimidinase-related protein 3 | 174.48 | -0.88 |
| Q76MZ3 | Serine/threonine-protein phosphatase 2A 65 kDa regulatory subunit A alpha isoform | 143.18 | -0.88 |
| P97427 | Dihydropyrimidinase-related protein 1 | 818.92 | -0.9 |
| P52480 | Pyruvate kinase PKM | 3457.2 | -0.9 |
| P39053 | Dynamin-1 | 1744.9 | -0.91 |
| Q9Z1B3 | 1-phosphatidylinositol 4,5-bisphosphate phosphodiesterase beta-1 | 41.48 | 10 mg/L |
| Q60597 | 2-oxoglutarate dehydrogenase, mitochondrial | 92.99 | 10 mg/L |
| Q5SSL4 | Active breakpoint cluster region-related protein | 51.4 | 10 mg/L |
| Q80Y20 | Alkylated DNA repair protein alkB homolog 8 | 129.16 | 10 mg/L |
| Q8C6Y6 | Ankyrin repeat and SOCS box protein 14 | 57.7 | 10 mg/L |
| O35643 | AP-1 complex subunit beta-1 | 60.21 | 10 mg/L |
| Q9DBG3 | AP-2 complex subunit beta | 110.45 | 10 mg/L |
| P06728 | Apolipoprotein A-IV | 180.65 | 10 mg/L |
| Q99KN1 | Arrestin domain-containing protein 1 | 175.26 | 10 mg/L |
| Q91VR2 | ATP synthase subunit gamma, mitochondrial | 185.97 | 10 mg/L |
| P97450 | ATP synthase-coupling factor 6, mitochondrial | 785.56 | 10 mg/L |
| Q9DC29 | ATP-binding cassette sub-family B member 6, mitochondrial | 32.46 | 10 mg/L |
| Q99PU8 | ATP-dependent RNA helicase DHX30 | 149.18 | 10 mg/L |
| Q9Z2H5 | Band 4.1-like protein 1 | 55.35 | 10 mg/L |
| Q9QYB8 | Beta-adducin | 84.36 | 10 mg/L |
| Q8BKX1 | Brain-specific angiogenesis inhibitor 1-associated protein 2 | 130.66 | 10 mg/L |
| P35762 | CD81 antigen | 627.76 | 10 mg/L |
| Q99N28 | Cell adhesion molecule 3 | 230.14 | 10 mg/L |
| A2A6Q5 | Cell division cycle protein 27 homolog | 59.73 | 10 mg/L |
| Q6A065 | Centrosomal protein of 170 kDa | 88.12 | 10 mg/L |
| A2A8L1 | Chromodomain-helicase-DNA-binding protein 5 | 40.82 | 10 mg/L |
| Q9EPU4 | Cleavage and polyadenylation specificity factor subunit 1 | 137.64 | 10 mg/L |
| Q8CDI7 | Coiled-coil domain-containing protein 150 | 73.6 | 10 mg/L |
| Q8CI04 | Conserved oligomeric Golgi complex subunit 3 | 29.61 | 10 mg/L |
| P19536 | Cytochrome c oxidase subunit 5B, mitochondrial | 392.36 | 10 mg/L |
| P48771 | Cytochrome c oxidase subunit 7A2, mitochondrial | 514.4 | 10 mg/L |
| Q8C4S8 | DENN domain-containing protein 2A | 64.83 | 10 mg/L |
| Q811D0 | Disks large homolog 1 | 78.4 | 10 mg/L |
| Q91XM9 | Disks large homolog 2 | 98.04 | 10 mg/L |
| Q62108 | Disks large homolog 4 | 206.56 | 10 mg/L |
| Q6PFD5 | Disks large-associated protein 3 | 52.24 | 10 mg/L |
| Q4U2R1 | E3 ubiquitin-protein ligase HERC2 | 88.09 | 10 mg/L |
| Q9DCS3 | Enoyl-[acyl-carrier-protein] reductase, mitochondrial | 119.84 | 10 mg/L |
| Q0VAV2 | Exophilin-5 | 38.17 | 10 mg/L |
| P47754 | F-actin-capping protein subunit alpha-2 | 227.89 | 10 mg/L |
| Q80X90 | Filamin-B | 32.25 | 10 mg/L |
| D3Z7P3 | Glutaminase kidney isoform, mitochondrial | 99.06 | 10 mg/L |
| P46425 | Glutathione S-transferase P 2 | 495.07 | 10 mg/L |
| Q9WV07 | Hydroperoxide isomerase ALOXE3 | 59.28 | 10 mg/L |
| Q3KNY0 | Immunoglobulin-like and fibronectin type III domain-containing protein 1 | 29.85 | 10 mg/L |
| Q571I4 | Inactive tyrosine-protein kinase PRAG1 | 58.94 | 10 mg/L |
| Q80V86 | Integrator complex subunit 8 | 71.24 | 10 mg/L |
| A6X935 | Inter alpha-trypsin inhibitor, heavy chain 4 | 42.89 | 10 mg/L |
| Q60625 | Intercellular adhesion molecule 5 | 48.95 | 10 mg/L |
| Q9QZ85 | Interferon-inducible GTPase 1 | 58.34 | 10 mg/L |
| Q62406 | Interleukin-1 receptor-associated kinase 1 | 142.9 | 10 mg/L |
| A2CG49 | Kalirin | 82.96 | 10 mg/L |
| P11369 | LINE-1 retrotransposable element ORF2 protein | 40.09 | 10 mg/L |
| Q9JI18 | Low-density lipoprotein receptor-related protein 1B | 39.31 | 10 mg/L |
| Q9JJ78 | Lymphokine-activated killer T-cell-originated protein kinase | 69.55 | 10 mg/L |
| O08539 | Myc box-dependent-interacting protein 1 | 320.05 | 10 mg/L |
| Q9DCT2 | NADH dehydrogenase [ubiquinone] iron-sulfur protein 3, mitochondrial | 107.77 | 10 mg/L |
| P52503 | NADH dehydrogenase [ubiquinone] iron-sulfur protein 6, mitochondrial | 194.46 | 10 mg/L |
| Q6GQX2 | Nck-associated protein 5-like | 147.69 | 10 mg/L |
| Q8R007 | Nectin-4 | 72.22 | 10 mg/L |
| P70211 | Netrin receptor DCC | 45.19 | 10 mg/L |
| Q9QXX8 | Nuclear fragile X mental retardation-interacting protein 1 | 98.96 | 10 mg/L |
| Q61937 | Nucleophosmin | 82.67 | 10 mg/L |
| O54998 | Peptidyl-prolyl cis-trans isomerase FKBP7 | 99.86 | 10 mg/L |
| Q5BL07 | Peroxisome biogenesis factor 1 | 140.96 | 10 mg/L |
| Q91YL7 | PGAP2-interacting protein | 52.95 | 10 mg/L |
| O70167 | Phosphatidylinositol 4-phosphate 3-kinase C2 domain-containing subunit gamma | 66.06 | 10 mg/L |
| O70250 | Phosphoglycerate mutase 2 | 112.61 | 10 mg/L |
| Q8CDG1 | Piwi-like protein 2 | 131.71 | 10 mg/L |
| O35129 | Prohibitin-2 | 402.09 | 10 mg/L |
| Q9QXV0 | ProSAAS | 113.13 | 10 mg/L |
| Q61207 | Prosaposin | 74.57 | 10 mg/L |
| Q8BHZ0 | Protein FAM49A | 149.49 | 10 mg/L |
| Q9CU24 | Protein THEMIS3 | 47.42 | 10 mg/L |
| Q8K183 | Pyridoxal kinase | 111.98 | 10 mg/L |
| P35294 | Ras-related protein Rab-19 | 293.2 | 10 mg/L |
| P24549 | Retinal dehydrogenase 1 | 132.8 | 10 mg/L |
| Q8BRH3 | Rho GTPase-activating protein 19 | 82.39 | 10 mg/L |
| Q8BWA8 | Rho guanine nucleotide exchange factor 19 | 263.26 | 10 mg/L |
| Q9CZC8 | Secernin-1 | 250.9 | 10 mg/L |
| B2RXR6 | Serine/threonine-protein phosphatase 6 regulatory ankyrin repeat subunit B | 116.3 | 10 mg/L |
| Q4VA53 | Sister chromatid cohesion protein PDS5 homolog B | 44.45 | 10 mg/L |
| Q3UHA3 | Spatacsin | 34.61 | 10 mg/L |
| Q8BWF0 | Succinate-semialdehyde dehydrogenase, mitochondrial | 188.93 | 10 mg/L |
| P61264 | Syntaxin-1B | 374.9 | 10 mg/L |
| A6H6E9 | Tetratricopeptide repeat protein 23-like | 78.77 | 10 mg/L |
| Q3UR70 | Transforming growth factor-beta receptor-associated protein 1 | 101.94 | 10 mg/L |
| Q00993 | Tyrosine-protein kinase receptor UFO | 51.64 | 10 mg/L |
| P56399 | Ubiquitin carboxyl-terminal hydrolase 5 | 68.64 | 10 mg/L |
| Q9DBP5 | UMP-CMP kinase | 91.98 | 10 mg/L |
| Q62059 | Versican core protein | 90.05 | 10 mg/L |
| P63024 | Vesicle-associated membrane protein 3 | 40.24 | 10 mg/L |
| P61161 | Actin-related protein 2 | 100.9 | 50 mg/L |
| Q9R0Q6 | Actin-related protein 2/3 complex subunit 1A | 103.13 | 50 mg/L |
| Q9WV32 | Actin-related protein 2/3 complex subunit 1B | 83.28 | 50 mg/L |
| Q9CVB6 | Actin-related protein 2/3 complex subunit 2 | 216.14 | 50 mg/L |
| Q9JM76 | Actin-related protein 2/3 complex subunit 3 | 574.56 | 50 mg/L |
| Q99JY9 | Actin-related protein 3 | 1211.34 | 50 mg/L |
| Q61271 | Activin receptor type-1B | 44.62 | 50 mg/L |
| Q9R0Y5 | Adenylate kinase isoenzyme 1 | 383.65 | 50 mg/L |
| P40124 | Adenylyl cyclase-associated protein 1 | 467.05 | 50 mg/L |
| Q920R0 | Alsin | 139.19 | 50 mg/L |
| Q7TQI7 | Ankyrin repeat and BTB/POZ domain-containing protein 2 | 93.72 | 50 mg/L |
| P84091 | AP-2 complex subunit mu | 254.17 | 50 mg/L |
| Q8BZ05 | Arf-GAP with Rho-GAP domain, ANK repeat and PH domain-containing protein 2 | 24.9 | 50 mg/L |
| Q497K5 | Arrestin domain-containing protein 5 | 80.25 | 50 mg/L |
| Q8R3P0 | Aspartoacylase | 61.32 | 50 mg/L |
| E9Q4N7 | AT-rich interactive domain-containing protein 1B | 21.9 | 50 mg/L |
| P12367 | cAMP-dependent protein kinase type II-alpha regulatory subunit | 204.71 | 50 mg/L |
| P31324 | cAMP-dependent protein kinase type II-beta regulatory subunit | 204.71 | 50 mg/L |
| B9EHT4 | CAP-Gly domain-containing linker protein 3 | 52.39 | 50 mg/L |
| Q5M8N0 | CB1 cannabinoid receptor-interacting protein 1 | 269.5 | 50 mg/L |
| Q91WS0 | CDGSH iron-sulfur domain-containing protein 1 | 189.08 | 50 mg/L |
| Q640L3 | Cell cycle progression protein 1 | 60.82 | 50 mg/L |
| Q7TSH4 | Centriolar coiled-coil protein of 110 kDa | 66.84 | 50 mg/L |
| Q62036 | Centrosomal protein of 131 kDa | 44.53 | 50 mg/L |
| E9Q309 | Centrosome-associated protein 350 | 26.11 | 50 mg/L |
| E9PZM4 | Chromodomain-helicase-DNA-binding protein 2 | 375.14 | 50 mg/L |
| Q80YR7 | Claspin | 89.03 | 50 mg/L |
| Q62425 | Cytochrome c oxidase subunit NDUFA4 | 1748.56 | 50 mg/L |
| O55071 | Cytochrome P450 2B19 | 63.07 | 50 mg/L |
| Q9CX98 | Cytochrome P450 2U1 | 59.31 | 50 mg/L |
| O35728 | Cytochrome P450 4A14 | 65.59 | 50 mg/L |
| Q99KU1 | Dehydrodolichyl diphosphate synthase complex subunit Dhdds | 105.52 | 50 mg/L |
| Q80U19 | Disheveled-associated activator of morphogenesis 2 | 38.52 | 50 mg/L |
| Q8K4R9 | Disks large-associated protein 5 | 54.05 | 50 mg/L |
| Q61881 | DNA replication licensing factor MCM7 | 53.8 | 50 mg/L |
| Q8CJ67 | Double-stranded RNA-binding protein Staufen homolog 2 | 90.3 | 50 mg/L |
| Q8R0K2 | E3 ubiquitin-protein ligase TRIM31 | 80.78 | 50 mg/L |
| P56564 | Excitatory amino acid transporter 1 | 143.9 | 50 mg/L |
| Q9WVH3 | Forkhead box protein O4 | 75.41 | 50 mg/L |
| Q8C0M0 | GATOR complex protein WDR59 | 31.1 | 50 mg/L |
| Q9ESZ8 | General transcription factor II-I | 115.83 | 50 mg/L |
| Q8CI94 | Glycogen phosphorylase, brain form | 37.32 | 50 mg/L |
| Q9ET01 | Glycogen phosphorylase, liver form | 25.65 | 50 mg/L |
| Q9WUB3 | Glycogen phosphorylase, muscle form | 25.65 | 50 mg/L |
| Q9CW79 | Golgin subfamily A member 1 | 86.47 | 50 mg/L |
| P55937 | Golgin subfamily A member 3 | 42.33 | 50 mg/L |
| Q91VW5 | Golgin subfamily A member 4 | 65.52 | 50 mg/L |
| Q8K349 | GTPase IMAP family member 6 | 69.55 | 50 mg/L |
| Q9R0C8 | Guanine nucleotide exchange factor VAV3 | 33.25 | 50 mg/L |
| P22361 | Hepatocyte nuclear factor 1-alpha | 72.1 | 50 mg/L |
| P61979 | Heterogeneous nuclear ribonucleoprotein K | 413.21 | 50 mg/L |
| Q8R081 | Heterogeneous nuclear ribonucleoprotein L | 125.22 | 50 mg/L |
| Q8VEK3 | Heterogeneous nuclear ribonucleoprotein U | 63.33 | 50 mg/L |
| O88569 | Heterogeneous nuclear ribonucleoproteins A2/B1 | 168.19 | 50 mg/L |
| Q91W97 | Hexokinase HKDC1 | 357.2 | 50 mg/L |
| O08528 | Hexokinase-2 | 344.36 | 50 mg/L |
| P70349 | Histidine triad nucleotide-binding protein 1 | 328.07 | 50 mg/L |
| Q8R366 | Immunoglobulin superfamily member 8 | 77.23 | 50 mg/L |
| Q61739 | Integrin alpha-6 | 56.16 | 50 mg/L |
| Q60677 | Integrin alpha-E | 55.64 | 50 mg/L |
| Q8BJD1 | Inter-alpha-trypsin inhibitor heavy chain H5 | 33.53 | 50 mg/L |
| Q9QY61 | Iroquois-class homeodomain protein IRX-4 | 116.02 | 50 mg/L |
| Q9QXL2 | Kinesin-like protein KIF21A | 71.51 | 50 mg/L |
| Q8C0R9 | Leucine-rich repeat and death domain-containing protein 1 | 50.31 | 50 mg/L |
| A2AWL7 | MAX gene-associated protein | 22.4 | 50 mg/L |
| Q8BJS8 | Mdm2-binding protein | 33.47 | 50 mg/L |
| B1AYB6 | Methyl-CpG-binding domain protein 5 | 130.49 | 50 mg/L |
| Q8K1A0 | Methyltransferase-like protein 5 | 88.52 | 50 mg/L |
| P54279 | Mismatch repair endonuclease PMS2 | 81.88 | 50 mg/L |
| Q99MD8 | Myoneurin | 59.94 | 50 mg/L |
| Q99LC3 | NADH dehydrogenase [ubiquinone] 1 alpha subcomplex subunit 10, mitochondrial | 298.33 | 50 mg/L |
| Q91V57 | N-chimaerin | 127.8 | 50 mg/L |
| Q810U3 | Neurofascin | 61.91 | 50 mg/L |
| P97460 | Neuronal PAS domain-containing protein 2 | 157.65 | 50 mg/L |
| Q61043 | Ninein | 34.85 | 50 mg/L |
| Q8R5A0 | N-lysine methyltransferase SMYD2 | 40.64 | 50 mg/L |
| Q02780 | Nuclear factor 1 A-type | 129.75 | 50 mg/L |
| P56716 | Oxygen-regulated protein 1 | 43.45 | 50 mg/L |
| Q9D4H9 | PHD finger protein 14 | 68.53 | 50 mg/L |
| P53811 | Phosphatidylinositol transfer protein beta isoform | 83.52 | 50 mg/L |
| G5E829 | Plasma membrane calcium-transporting ATPase 1 | 93.23 | 50 mg/L |
| Q9R0K7 | Plasma membrane calcium-transporting ATPase 2 | 77.91 | 50 mg/L |
| P26618 | Platelet-derived growth factor receptor alpha | 48.55 | 50 mg/L |
| Q6TDU8 | Protein CASC1 | 29.28 | 50 mg/L |
| Q2EMV9 | Protein mono-ADP-ribosyltransferase PARP14 | 33.61 | 50 mg/L |
| Q3UYC0 | Protein phosphatase 1H | 136.49 | 50 mg/L |
| E5FYH1 | Protein TOPAZ1 | 46.16 | 50 mg/L |
| P35486 | Pyruvate dehydrogenase E1 component subunit alpha, somatic form, mitochondrial | 75.99 | 50 mg/L |
| G3UYX5 | Regulator of G-protein signaling 22 | 61.47 | 50 mg/L |
| Q9ES97 | Reticulon-3 | 88.22 | 50 mg/L |
| P70336 | Rho-associated protein kinase 2 | 37.51 | 50 mg/L |
| Q9D7H3 | RNA 3'-terminal phosphate cyclase | 151.46 | 50 mg/L |
| A2AGL3 | Ryanodine receptor 3 | 31.45 | 50 mg/L |
| Q91V61 | Sideroflexin-3 | 115.13 | 50 mg/L |
| Q923Q2 | StAR-related lipid transfer protein 13 | 72.57 | 50 mg/L |
| Q99JB2 | Stomatin-like protein 2, mitochondrial | 113.32 | 50 mg/L |
| Q9ERG2 | Striatin-3 | 44.66 | 50 mg/L |
| Q9Z2I9 | Succinate--CoA ligase [ADP-forming] subunit beta, mitochondrial | 44.71 | 50 mg/L |
| Q8CHC4 | Synaptojanin-1 | 51.04 | 50 mg/L |
| Q5SVR0 | TBC1 domain family member 9B | 32.16 | 50 mg/L |
| Q7TN22 | Thioredoxin domain-containing protein 16 | 86.01 | 50 mg/L |
| P70399 | TP53-binding protein 1 | 100.25 | 50 mg/L |
| P42669 | Transcriptional activator protein Pur-alpha | 216.84 | 50 mg/L |
| Q9R1Q8 | Transgelin-3 | 312.49 | 50 mg/L |
| P24529 | Tyrosine 3-monooxygenase | 133.86 | 50 mg/L |
| Q7TQI3 | Ubiquitin thioesterase OTUB1 | 613.88 | 50 mg/L |
| A2RSJ4 | UHRF1-binding protein 1-like | 334.57 | 50 mg/L |
| Q3U2A8 | Valine--tRNA ligase, mitochondrial | 33.7 | 50 mg/L |
| Q8K1X1 | WD repeat-containing protein 11 | 52.43 | 50 mg/L |

ªUniprot accession ID retrieved from uniport.org database; Negative values of fold change mean down-regulated proteins; 10 mg/L or 50 mg/L in fold change column means that protein was exclusively found in the respective experimental
